# Engineering *Haloferax mediterranei* as an Efficient Platform for High Level Production of Lycopene

**DOI:** 10.3389/fmicb.2018.02893

**Published:** 2018-11-29

**Authors:** Zhen-Qiang Zuo, Qiong Xue, Jian Zhou, Da-He Zhao, Jing Han, Hua Xiang

**Affiliations:** ^1^State Key Laboratory of Microbial Resources, Institute of Microbiology, Chinese Academy of Sciences, Beijing, China; ^2^College of Life Sciences, University of Chinese Academy of Sciences, Beijing, China

**Keywords:** lycopene, biosynthesis, *Haloferax mediterranei*, rate-limiting steps, phytoene synthase, phytoene desaturase, bacterioruberin, PHBV

## Abstract

Lycopene attracts increasing interests in the pharmaceutical, food, and cosmetic industries due to its anti-oxidative and anti-cancer properties. Compared with other lycopene production methods, such as chemical synthesis or direct extraction from plants, the biosynthesis approach using microbes is more economical and sustainable. In this work, we engineered *Haloferax mediterranei*, a halophilic archaeon, as a new lycopene producer. *H. mediterranei* has the *de novo* synthetic pathway for lycopene but cannot accumulate this compound. To address this issue, we reinforced the lycopene synthesis pathway, blocked its flux to other carotenoids and disrupted its competitive pathways. The reaction from geranylgeranyl-PP to phytoene catalyzed by phytoene synthase (CrtB) was identified as the rate-limiting step in *H. mediterranei*. Insertion of a strong promoter P_phaR_ immediately upstream of the *crtB* gene, or overexpression of the heterologous CrtB and phytoene desaturase (CrtI) led to a higher yield of lycopene. In addition, blocking bacterioruberin biosynthesis increased the purity and yield of lycopene. Knock-out of the key genes, responsible for poly(3-hydroxybutyrate-*co*-3-hydroxyvalerate) (PHBV) biosynthesis, diverted more carbon flux into lycopene synthesis, and thus further enhanced lycopene production. The metabolic engineered *H. mediterranei* strain produced lycopene at 119.25 ± 0.55 mg per gram of dry cell weight in shake flask fermentation. The obtained yield was superior compared to the lycopene production observed in most of the engineered *Escherichia coli* or yeast even when they were cultivated in pilot scale bioreactors. Collectively, this work offers insights into the mechanism involved in carotenoid biosynthesis in haloarchaea and demonstrates the potential of using haloarchaea for the production of lycopene or other carotenoids.

## Introduction

Lycopene is a C40 isoprenoid compound in the carotenoid family. Due to its anti-oxidative and anti-cancer activities (Sies and Stahl, 1998; Gajowik and Dobrzynska, 2014), lycopene has been widely used for nutritional supplements, pharmaceutical and cosmetic products (Wei et al., 2017). The conventional methods for lycopene production include direct extraction from plants,chemical synthesis and microbial fermentation. Among these methods, microbial production of lycopene is more economical and sustainable (Chen et al., 2016). Recently, with the development of metabolic engineering techniques and synthetic biology, lycopene overproduction has been realized in *Escherichia coli* (Zhu et al., 2015; Wei et al., 2017; Wu et al., 2018; Xu et al., 2018), yeast (Xie et al., 2015a; Schwartz et al., 2017), *Blakeslea trispora* (Liu et al., 2012; Wang et al., 2016, 2017), and *Rhodobacter sphaeroides* (Su et al., 2018). However, the field is seeking a better platform for large-scale production of lycopene or other carotenoids. Halophilic archaea (haloarchaea) belong to the domain Archaea and are unique microorganisms that survive under the high salt condition (Singh and Singh, 2017). Many haloarchaeal species are capable of producing the compounds of the carotenoid family (Rodrigo-Banos et al., 2015), such as phytoene, β-carotene, lycopene, as well as the derivatives of bacterioruberin and salinixanthin (de Lourdes Moreno et al., 2012). Particularly, they hold several advantages for carotenoid production: the high-salt tolerance enables haloarchaea cultivation under non-sterile condition and thus reduces the energy cost (Singh and Singh, 2017). Additionally, the process of carotenoid extraction from haloarchaea is relatively simple, as the cell lysis undergoes in low sodium chloride (NaCl) condition. Consequently, haloarchaea are considered as an alternative producer for carotenoids (Naziri et al., 2014).

*Haloferax mediterranei* can use probably the largest range of single carbon sources and grows faster than other known members of the *Halobacteriaceae* (Oren and Hallsworth, 2014). Its complete genome information is available (Han et al., 2012), and the *pyrF*-based gene knockout system for genome-wide manipulation has also been well-established in this strain (Liu et al., 2011). With these merits, *H. mediterranei* has been one of the most common model strains for the study of physiology and metabolism in archaea. For example, it has been used to investigate poly(3-hydroxybutyrate-*co*-3-hydroxyvalerate) (PHBV) biosynthesis and its metabolism regulation processes (Han et al., 2013, 2017; Zhao et al., 2013; Bhattacharyya et al., 2014; Cai et al., 2015). However, only a few studies on its carotenoid production has been reported till recently. Fang et al. (2010) improved the C50 carotenoid production to 0.604 A_494__nm_/mL broth *via* a two-stage cultivation approach. Chen et al. (2015) used extruded rice bran and starch under optimal conductivity of brined medium for a high red pigment production of 556 mg/L. However, there is no work investigating either the production of other carotenoids (e.g., lycopene), or the pathway engineering to improve carotenoid production in *H. mediterranei*.

**FIGURE 1:**
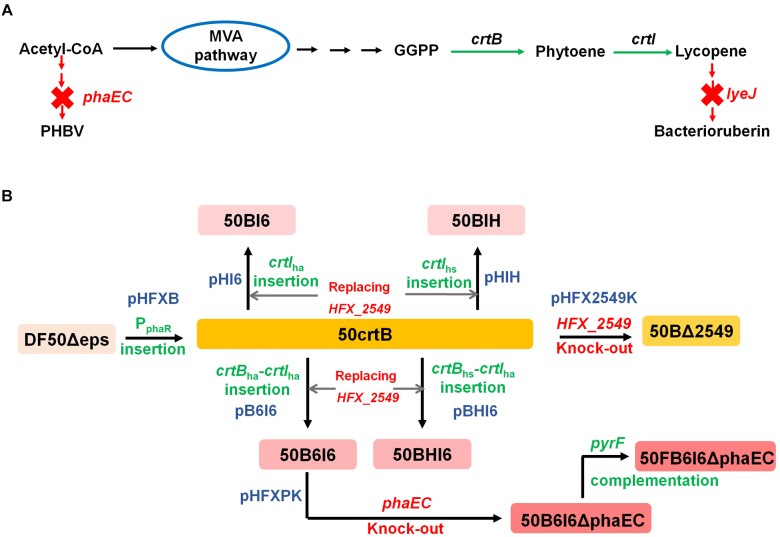
Schematic illustration of engineered *H. mediterranei* for lycopene production. **(A)** Enhancement of lycopene synthesis by enhancing lycopene biosynthetic pathway and blocking its flux to bacterioruberin and its competitive pathway for PHBV biosynthesis. Green arrows represent enhanced steps and red arrows and crosses represent blocked steps. **(B)** Flowchart of engineering *H. mediterranei* strain for increasing lycopene synthesis. Green terms represent gene insertion and red ones represent gene deletion. The blue terms show the plasmids used for constructing the mutants of *H. mediterranei*.

In this study, we explored the possibility to use *H. mediterranei* as a potential cell factory for lycopene production by multiple strategies (Figure 1). First, we attempted to identify and eliminate the rate-limiting steps involved in lycopene biosynthesis. Then we disrupted bacterioruberin synthesis to increase lycopene accumulation and purity. Heterologous phytoene synthase (CrtB) and phytoene desaturase (CrtI) encoding genes from other haloarchaea were employed for further enhancing lycopene production. Subsequently, we blocked PHBV synthesis to divert more acetyl-CoA flux to lycopene synthesis and also complemented the *pyrF* gene in the engineered strain for its future application in industrial scale. We finally obtained a metabolic engineered *H. mediterranei* strain with relatively high purity and production of lycopene.

## Materials and Methods

### Strains, Medium, and Culture Conditions

All the strains used in this study are listed in Supplementary Table S1. *E. coli* JM109 (Sambrook, 1989) was used for plasmids construction and *E. coli* JM110 was used to eliminate the methylated plasmid *in vivo* (Palmer and Marinus, 1994). Luria Broth (LB) medium was used for *E. coli* culture at 37°C. When needed, 100 μg/mL of ampicillin was added to LB medium. *H. mediterranei* was cultivated at 37°C in nutrient-rich AS-168 medium (per liter, 5 g casamino acids, 5 g yeast extract, 1 g sodium glutamate, 3 g trisodium citrate, 2 g KCl, 20 g MgSO_4_ ⋅ 7H_2_O, 200 g NaCl, 5 mg FeSO_4_ ⋅ 7H_2_O, and 0.036 mg MnCl_2_ ⋅ 4H_2_O [pH 7.0]). AS-168SY medium was similar to AS-168 medium, except that yeast extract was excluded. Plasmids were transformed into *H. mediterranei* with the polyethylene glycol-mediated transformation method (Cline et al., 1989). When required, AS-168 medium was supplemented with 50 mg/mL uracil (Sangon, China) and 250 mg/mL 5-Fluoroorotic acid (Sangon, China) for counter-selection of the recombinants without *pyrF* marker. For lycopene production, a 1% (V/V) seed culture of *H. mediterranei* or its mutants was inoculated into a shake flask containing 50 mL of MG medium (per liter, 110 g NaCl, 20.51 g MgCl_2_, 29.52 g MgSO_4_, 5 g KCl, 1 g CaCl_2_, 2 g NH_4_Cl, 0.0375 g KH_2_PO_4_, 10 g glucose, 15 g PIPES, Fe(III) citrate, and 1 mL trace element solution SL-6 [pH 7.2]) (Antón et al., 1988) and cultured at 37°C and 200 rpm for 7 days.

### Plasmid Construction for Gene Overexpression

The native candidate genes involved in lycopene synthesis were amplified with primers listed in Supplementary Table S2 from the *H. mediterranei* genomic DNA. The heterologous *crt* genes *HAH_2563* (*crtB*_ha_), *HAH_1058* (*crtI*_ha_) and *OE_3093R* (*crtB*_hs_), *OE_3381R* (*crtI*_hs_) were obtained *via* PCR from the genomic DNA of *Haloarcula hispanica* and *Halobacterium salinarum*, respectively. Amplified fragments were inserted into pWLR [derived from pWL502 by insertion of a strong promoter P_phaR_ (Cai et al., 2012)] digested with BamHI and XbaI, by using One Step Cloning Kit (Yeasen, Co., Ltd., China), to generate plasmids for gene overexpression under the control of promoter P_phaR_ (Supplementary Table S1).

### Plasmid Construction for Gene Integration in Chromosome

All plasmids for gene knock-in or knock-out were constructed based on a suicide plasmid pHFX (Liu et al., 2011). A 583-bp DNA fragment located immediately upstream of *crtB*_hm_ was amplified with primer pair *crtB*-in-1/*crtB*-in-2 from *H. mediterranei* genomic DNA. Another 580-bp fragment containing promoter P_phaR_ and the partial 5′ region of *crtB* was amplified with primer pair *crtB*-in-3/*crtB*-in-4 from plasmid pW2547 used for *crtB*_hm_ overexpression (Supplementary Table S1). Then, the two PCR products were inserted into the plasmid pHFX to construct the integration plasmid of pHFXB, which was used to replace the native promoter of *HFX_2547 (crtB*_hm_) in chromosome. For the heterologous *crt* gene integration, a 524-bp fragment up-stream of *HFX_2549* and a 529-bp fragment down-stream of *HFX_2549* were amplified by relevant primer pairs (Supplementary Table S2). Different *crt* gene fragments containing P_phaR_ were amplified by using *crtB*_ha_, *crtI*_ha_, *crtB*_hs_, and *crtI*_hs_ overexpression plasmids pWHA2563, pWHA1058, pWOE3093, and pWOE3381 (Supplementary Table S1) as PCR template, respectively. The corresponding homologous arm fragments and different *crt* genes, containing promoter P_phaR_, were assembled into pHFX to construct plasmids containing different *crt* genes used for their integration in chromosome (Supplementary Figure S1 and Supplementary Table S1). Similar to the plasmid construction method as described above, primer pairs *HFX_2549*-K1/*HFX_2549*-K2 and *HFX_2549*-K3/*HFX_2549*-K4 were used to construct the plasmid pHFX2549K for *HFX_2549* knock-out. Primer pairs *phaEC*-K1/*phaEC*-K1 and *phaEC*-K3/*phaEC*-K4 were used for the construction of pHFXPK for *phaEC* knock-out (Supplementary Table S1).

### Analysis of Carotenoids by Thin Layer Chromatography (TLC)

Carotenoids in acetone extract obtained from different cultures were analyzed by thin layer chromatography (TLC), following the protocol described by Strand et al. (1997) with slight modifications. Briefly, after cultivation in MG medium for 7 days, the cells (1 mL) were harvested by centrifugation (12,000 × *g* for 5 min, at 4°C), and resuspended in acetone (1 mL) under a reduced light condition to prevent photo-bleaching and degradation (Alper and Stephanopoulos, 2008). The acetone supernatant containing carotenoids was collected and transferred to a new tube. This process was repeated until the pellets were totally white. Acetone extracted carotenoids (10 μL) were analyzed by TLC on a silica plate (GF254, Qingdao Haiyang Chemical, Co., Ltd., China) with acetone and n-heptane (1:1, v/v) as the development liquid in fume hood at room temperature. In addition, the visualized spot on the resulting TLC plate was scraped off, and extracted with 200 μL of acetone. The obtained supernatant was then scanned under 350–550 nm.

### Lycopene Quantification

The lycopene content in the extract was determined by using a HPLC system (Agilent, 1260, United States) equipped with a ZORBAX Eclipse XDB-C18 column (4.6 mm × 150 mm, 5 μm) and a UV/VIS detector. The absorption was detected at 450 nm. The mobile phase consisted of methanol-isopropanol (65:35 V/V) with a flow rate of 1 mL/min at 30°C. Injection volume of sample was 20 μL. The lycopene concentration was calculated based on the calibration curve of lycopene (Macklin Biochemical, Co., Ltd., China).

### PHA Content Analysis

The cells were collected by centrifugation at 10,000 × *g*, 4°C, 15 min and lyophilized. The lyophilized cells were treated with a mixture of chloroform and methanol containing 3% (v/v) sulfuric acid at 95°C for 4 h. The resulting hydroxyacyl methylesters were then analyzed by GC-6820 instrument (Agilent, United States) as described by Han et al. (2007).

### RNA Extraction and Quantitative Reverse Transcription-PCR (qRT-PCR)

The cells were cultured in AS-168 medium at 37°C for 12 h and subsequently harvested by centrifugation (12,000 × *g*, 4°C). The total RNA was extracted using TRIzol reagent (Invitrogen, United States) as previously described (Lu et al., 2008a). TURBO DNA-free^TM^ Kit (Thermo Fisher Scientific, United States) was used for removing DNA contamination. The cDNA was synthesized by reverse transcription with random hexamer primers from 1 μg of DNA-free total RNA using the Moloney Murine Leukemia Virus Reverse Transcriptase (M MLV-RT) (Promega, United States). The relative fold of gene expression was analyzed by ViiA^TM^ 7 Real-Time PCR System (Applied Biosystems, Inc., United States), using 7S RNA as an endogenous control to normalize the data of each sample. The primers used are listed in Supplementary Table S2.

### Sequences Analysis and Databases

The DNA sequences were obtained from National Center for Biotechnology Information (NCBI) Genome Database. The information about the most identified enzymes involved in MVA and lycopene synthesis pathway (supported by evidence at protein level), was accessed from UniProt Database^1^. Sequence homology was assessed by BLASTN or BLASTP in NCBI (Altschul et al., 1990). Predictions of transmembrane helices in the proteins were performed by using the TMHMM Server v2.0^2^ (Krogh et al., 2001).

The genome accession numbers deposited in GenBank are as following, *H. mediterranei* (CP001868.2), *H. hispanica* (NC_015948.1), and *H. salinarum* (AM774415.1).

### Statistical Analysis

Experiments were performed in triplicate and data was analyzed by the GraphPad Prism 7 software and represented as mean ± standard deviation. Statistical analysis was done using a two-tailed *t*-test. Statistical significance was defined as ^∗^*p* < 0.05.

## Results

### Identifying the Rate-Limiting Steps Involved in Native Lycopene Biosynthesis

**FIGURE 2:**
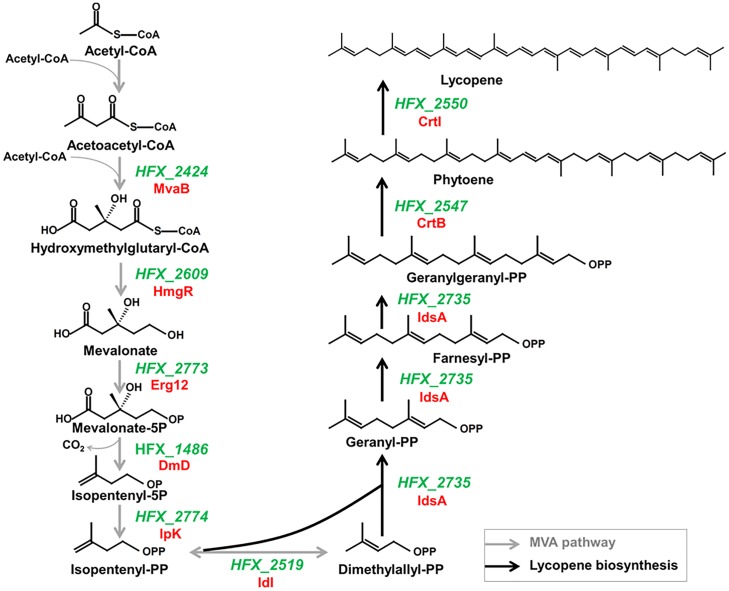
Proposed main steps in the mevalonate and lycopene biosynthesis pathways of *H. mediterranei* based on the KEGG pathway database. For simplicity, cofactors and ATP consumption are not shown. Gray arrows show the MVA pathway and black ones show the lycopene biosynthetic pathway descripted in this study.

*In silico* metabolic pathway analysis reveals that *H. mediterranei* has a complete lycopene biosynthetic pathway, referring to the steps from isopentenyl-PP (IPP) and dimethylallyl-PP (DMAPP) to lycopene (Figure 2). Mevalonate (MVA) pathway provides the two important precursors, IPP and DMAPP, for lycopene synthesis. In MVA pathway, two molecules of acetyl-CoA are condensed to form acetoacetyl-CoA and a third acetyl-CoA molecule is then added to form 3-hydroxy-3-methylglutaryl-CoA (HMG-CoA) by Hydroxymethylglutaryl-CoA synthase (MvaB, HFX_2424). The next step involves the conversion of HMG-CoA to MVA by Hydroxymethylglutaryl-CoA reductase (HmgR, HFX_2609). MVA is then phosphorylated by Mevalonate kinase (Erg12, HFX_2773) to generate mevalonate phosphate (MVAP). Different from the classical MVA pathway, an alternative pathway is proposed for the IPP generation from MVAP, which is catalyzed by Diphosphomevalonate decarboxylase (DmD, HFX_1486) and Isopentenyl phosphate kinase (IpK, HFX_2774). IPP can be then isomerized to DMAPP by Isopentenyl-diphosphate delta-isomerase (IdI, HFX_2519). DMAPP and IPP are condensed to geranyl-PP (GPP) and then to farnesyl-PP (FPP) and finally to geranylgeranyl-PP (GGPP) by trifunctional prenyl diphosphate synthase (IdsA, HFX_2735). Two molecules of GGPPs are condensed by CrtB (HFX_2547) to form phytoene and then it undergoes four consecutive desaturation reactions catalyzed by CrtI (HFX_2550) to produce lycopene.

**FIGURE 3:**
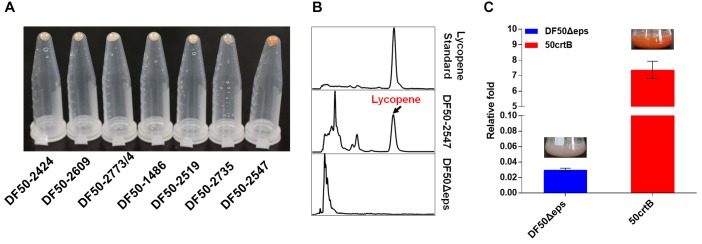
Identification of the rate-limiting steps involved in lycopene biosynthesis in *H. mediterranei*. **(A)** Collected *H. mediterranei* cells (1 mL) with a single gene overexpressed in eppendorf tubes. *HFX_2773* and *HFX_2774* are overlapped by four nucleotides and thus they are co-expressed. **(B)** HPLC analysis of the lycopene produced by DF50-2547 and DF50Δeps. **(C)** qRT-PCR analysis of *crtB*_hm_ transcriptional level in DF50Δeps and 50crtB. The relative fold of *crtB*_hm_ gene expression level is calculated by normalization to the expression of inner control 7S RNA. Insets are the photographs of 7-day shake flask cultures.

Although *H. mediterranei* possessed the complete lycopene synthetic pathway, we could not detect lycopene accumulation in this strain (Figure 3B). The extremely low lycopene production might be due to the rate-limiting steps in its lycopene biosynthesis. According to the previous studies about rate-limiting steps in MVA and lycopene synthesis pathway (Goldstein and Brown, 1990; Kang et al., 2005; Steussy et al., 2005; Anthony et al., 2009; Lombard and Moreira, 2011; Berthelot et al., 2012), we selected all the predicted genes as our candidates to be overexpressed in *H. mediterranei* (Figure 2 and Table 1). To rapidly identify the rate-limiting steps, we used a plasmid-based expression system for candidate genes overexpression under the control of a strong constitutive promoter P_phaR_. The expression plasmid containing each gene was transformed into the strain DF50Δeps (Zhao et al., 2013) individually and correct transformants were confirmed by PCR and Sanger sequencing. Functional overexpression of the genes encoding rate-limiting enzymes made transformants orange or even red, so it was easy to identify the genes encoding rate-limiting enzymes. Obviously, only the overexpression of gene *crtB*_hm_ resulted in an orange colored phenotype (Figure 3A). Meanwhile the lycopene accumulation in this *crtB*_hm_-overexpressed strain DF50-2547 was further confirmed by HPLC (Figure 3B), whereas no lycopene accumulation was detected in other gene overexpressed strains (Supplementary Figure S2). These results indicated that the step from GGPP to phytoene was the rate-limiting step in lycopene synthesis in *H. mediterranei*.

**Table 1:** Identity analysis between candidate enzymes involved in lycopene biosynthesis in *H. mediterranei* and idendified enzymes.

Enzyme in *H. mediterranei*	Identified enzymes	Protein identity
Enzyme	Gene	Gene	Reference strain	
MvaB	*HFX_2424*	*HVO_2419*	*H. vocanii* (VanNice et al., 2013)	95%
HmgR	*HFX_2609*	*HVO_2583*	*H. vocanii* (Bischoff and Rodwell, 1996)	89%
Erg12	*HFX_2773*	*HVO_2761*	*H. vocanii* (Azami et al., 2014)	92%
DmD	*HFX_1486*	*HVO_1412*	*H. vocanii* (VanNice et al., 2014)	90%
IpK	*HFX_2774*	*HVO_2762*	*H. vocanii* (VanNice et al., 2014)	89%
IdI	*HFX_2519*	*idi*	*E. coli* (Hemmi et al., 1998)	32%
IdsA	*HFX_2735*	*ispA*	*E. coli* (Hosfield et al., 2004)	31%
		*crtE*	*E. vulneris*	30%
CrtB	*HFX_2547*	*crtB*	*Synechococcus elongatus* (Chamovitz et al., 1992)	32%
CrtI	*HFX_2550*	*crtI*	*Erwinia uredovora* (Fraser et al., 1992)	31%

### Reinforcing the Rate-Limiting Step in Lycopene Synthesis by Insertion of a Strong Promoter

The plasmid-based overexpression of *crtB*_hm_ could reinforce the rate-limiting step and thus enhanced the production of lycopene in *H. mediterranei*. However, this plasmid-based system is not genetically stable and probably brings a metabolic burden. To address this issue, we therefore constructed the plasmid pHFXB (Supplementary Table S1) and used a two-step homologous recombination method to insert promoter P_phaR_ into the chromosome immediately up-stream of *crtB*_hm_ in DF50Δeps. The engineered strain, termed 50crtB, was easy to be distinguished visually, because of its orange color, which was different from the light pink color of its parental strain DF50Δeps (Figure 3C).

**FIGURE 4:**
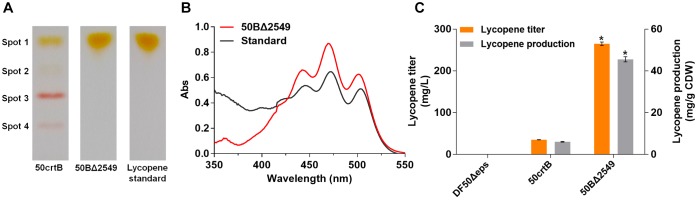
Analysis of lycopene production of 50crtB and 50BΔ2549. **(A)** TLC analysis of the carotenoids extracted from 50crtB and 50BΔ2549. Spot 1 contains mainly lycopene, Spot 2–Spot 4 consists of bacterioruberin and its derivates. **(B)** UV-Vis absorption spectra of the carotenoids extracted from the pigment spot of 50BΔ2549 in TLC plate. The wavelength range is set as 350 ∼ 550 nm. **(C)** Lycopene titer and production of 50crtB and 50BΔ2549. Strains are cultured for 7 days in 50 mL MG medium at 37°C in shake flasks. *^∗^p* < 0.05, lycopene production and titer of the group 50BΔ2549 are compared with the 50crtB. Data is expressed as Mean ± SD of triplicate determinations.

Next, we analyzed the transcription level of *crtB*_hm_ in strain 50crtB and DF50Δeps by qRT-PCR. The result showed that the insertion of promoter P_phaR_ dramatically increased the transcription level of *crtB*_hm_ by 245 times, when compared with the DF50Δeps strain (Figure 3C). As expected, the high CrtB**_hm_ expression level significantly promoted the conversion from GGPP to phytoene and subsequently improved lycopene biosynthesis. After cultivation in shake flasks containing 50 mL MG medium for 7 days, a lycopene production of 6.05 ± 0.18 mg/g dry cell weight (DCW) (35.15 ± 0.43 mg/L) was achieved (Figure 4C).

### Disrupting Bacterioruberin Biosynthesis to Improve the Accumulation and Purity of Lycopene

Lycopene is the last shared intermediate in bacterioruberin and retinal biosynthesis in some haloarchaea (Peck et al., 2017). However, *H. mediterranei* lacks the genes, *crtY* (encoding lycopene cyclase) (Peck et al., 2002), *brp* and *blh* (encoding β-carotene dioxygenase) (Peck et al., 2001), involved in the retinal biosynthesis pathway and therefore, lycopene can only flux into bacterioruberin biosynthesis (Figure 1A). Thus, we next blocked bacterioruberin biosynthesis to enhance lycopene accumulation and its purity. Bioinformatic analysis revealed the presence of two genes (*HFX_1501* and *HFX_2549*) potentially involved in bacterioruberin synthesis in *H. mediterranei*. Both of them are annotated as putative prenyltransferases, which can transfer 5-carbon prenyl groups to various substrates. *HFX_1501* encodes a protein of 284 amino acid residues, which showed 30 and 28% identity to the LyeJ of *H. salinarum* and *H. japonica*, respectively (Supplementary Figure S3A). In the case of *HFX_2549*, it encodes a 292-amino acid protein exhibiting 64 and 61% identity to the LyeJ of *H. salinarum* and *H. japonica*, respectively (Supplementary Figure S3B). Moreover, it is located within the carotenoid biosynthetic gene cluster. Furthermore, a membrane topology analysis using TMHMM revealed that *HFX_2549* encoded an integral membrane protein containing seven transmembrane domains (Supplementary Figure S3B), consistent with the LyeJ of *H. salinarum* or *H. japonica*. This suggested that *HFX_2549* is likely to encode LyeJ in *H. mediterranei.*

We knocked out the gene, *HFX_2549*, and obtained the strain 50BΔ2549. The carotenoid components of the 50crtB and 50BΔ2549 strains were analyzed qualitatively by TLC. The sample from 50crtB displayed multiple spots on the silica plate (Figure 4A, Spots 1–4), which represented lycopene, bacterioruberin and its derivates. In contrast, the sample from 50BΔ2549 only contained a single spot 1 (Figure 4A, Spot 1). Subsequently, spot 1 on the silica plate from the 50BΔ2549 strain was recovered and extracted by using acetone. The UV-Vis absorbance spectrum of the extracted sample had a typical three-finger shape of lycopene at 442, 470 and 501 nm, similar to the absorption spectrum of lycopene standard (Figure 4B). These results indicated that 50BΔ2549 could not synthesize bacterioruberin and its derivates, and the reaction was terminated at the lycopene step. Consequently, HFX_2549 was the key enzyme for bacterioruberin synthesis in *H. mediterranei*. Lycopene production of 50BΔ2549 was further quantified by HPLC. The production significantly increased to 45.54 ± 1.23 mg/g DCW, which was about 6.5 times higher than that of 50crtB (Figure 4C).

### Lycopene Production Improvement by Importing Phytoene Desaturase From Other Haloarchaea

**FIGURE 5:**
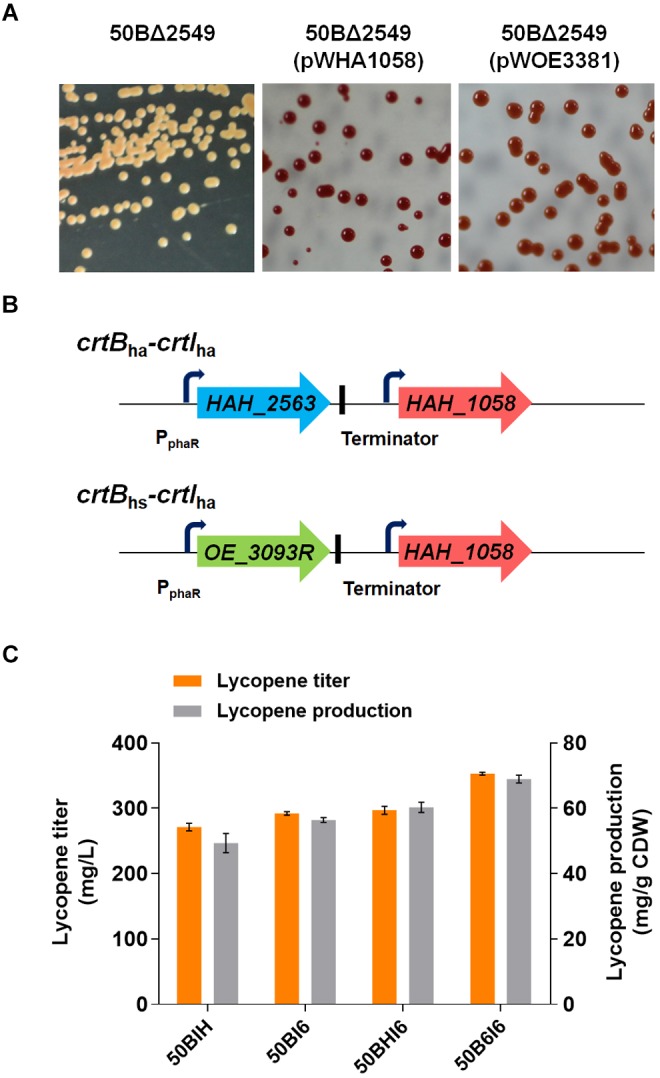
Importation of heterologous *crt* genes to improve lycopene production. **(A)** Colony color of *H. mediterranei* 50BΔ2549 cultured in AS-168 medium and the strains after introducing the expression plasmid containing *crtI* from *Haloarcula hispanica* or *Halobacterium salinarum* cultured in AS-168Y medium. 50BΔ2549 with pWHA1058 means *crtI*_ha_ expressed strain and pWOE3381 means *crtI*_hs_ expressed strain. **(B)** *crtB-crtI* expression cassettes. Each gene was under the control of P_phaR_ promoter and a terminator was in the downstream of *crtB*_ha_ or *crtB*_hs_. **(C)** Lycopene titer and production of engineered *H. mediterranei* by insertion of heterologous *crt* genes in the chromosome of 50crtB by replacing *HFX_2549*. Strains are cultured for 7 days in 50 mL MG medium at 37°C in shake flasks.

To improve the yield of the target products, it is often necessary to enhance the availability of essential precursors (Xie et al., 2015a). In our study, overexpression of *crtB*_hm_ supplied more precursor, phytoene, for lycopene synthesis, and enhancement of the flux from phytoene to lycopene is another strategy for lycopene production improvement. Here, we strengthened the process by importing heterologous gene *crtI*_ha_ or *crtI*_hs_ from carotenogenic haloarchaea, *H. hispanica* or *H. salinarum*. First, we confirmed the function of *crtI*_ha_ and *crtI*_hs_ by transforming their expression plasmid pWHA1058 or pWOE3381 (Supplementary Table S1) into the 50BΔ2549 strain. The positive transformants with the functional expression of heterologous *crtI* showed enhanced color intensity (Figure 5A). Afterward, we constructed two plasmids, pHI6 and pHIH (Supplementary Table S1), for the integration of *crtI*_ha_ and *crtI*_hs_ into the chromosome of *H. mediterranei* (Supplementary Figure S1A). To avoid unpredictable effects brought by the insertion site, we used 50crtB as the host and integrated these two genes separately in the chromosome by replacing *HFX_2549* (Figure 1B). The two resultant strains were named 50BI6 and 50BIH (Supplementary Table S1).

Lycopene production by the engineered strains were then determined by HPLC. Lycopene yields of 56.46 ± 0.74 mg/g DCW (292.21 ± 2.88 mg/L) and 49.37 ± 2.95 mg/g DCW (271.43 ± 6.07 mg/L) were obtained in the engineered strains, 50BI6 and 50BIH, respectively. This result showed that the heterologous expression of *crtI*_ha_ in *H. mediterranei* was more effective for enhancing lycopene synthesis compared to *crtI*_hs_. Finally, 50BI6 got a 24.0% increase in lycopene production and 10.2% increase in lycopene titer compared to 50BΔ2549 (Figure 5C).

### Heterologous *crtB* Overexpression for Further Optimizing Lycopene Production

The high lycopene production strain 50BI6 contained two copies of *crtI* (*crtI*_hm_ and *crtI*_ha_). To investigate whether CrtB was still the rate-limiting enzyme in 50BI6, another copy of *crtB* from *H. hispanica* or *H. salinarum* was introduced into 50BI6. We constructed two types of *crtB*-*crtI* expression cassettes, *crtB_ha_-crtI*_ha_ and *crtB_hs_-crtI*_ha_ (Figure 5B) and inserted these expression cassettes in the chromosome of 50crtB by replacing *HFX_2549* to obtain new strains, 50B6I6 and 50BHI6 (Figure 1B and Supplementary Figure S1B). We analyzed the transcriptional status of *crtB*_ha_, *crtB*_hs_, and *crtI*_ha_ in these two strains and found that all the expected genes were successfully transcribed base on the RT-PCR analysis (Supplementary Figure S4). The lycopene yields of 50B6I6 and 50BHI6 were further increased to 68.95 ± 1.19 mg/g DCW (353.06 ± 2.39 mg/L) and 60.33 ± 1.56 mg/g DCW (297.05 ± 6.50 mg/L), respectively (Figure 5C). This suggested that the strains with co-introduced *crtB*_ha_ and *crtI*_ha_ could produce more lycopene. Lycopene titer displayed a 20.8% increase in 50B6I6 compared to 50BI6.

### Disruption of PHBV Biosynthesis to Divert More Acetyl-CoA Flux to Lycopene

*H. mediterranei* can accumulate a large amount of PHBV when cultured in MG medium (Zhao et al., 2013) and acetyl-CoA is an important precursor for its biosynthesis (Don et al., 2006). Blocking acetyl-CoA flux to PHBV biosynthesis may be able to further maximize the lycopene production (Figure 1B). To prove this, we knocked out the PHBV synthase encoding genes, *phaE and phaC* (Lu et al., 2008b), in 50B6I6 and obtained a new strain, named 50B6I6ΔphaEC (Supplementary Table S1). It could not produce PHBV as determined by gas chromatography (Table 2) and this result suggested that the deletion of *phaEC* blocked PHBV synthesis. Moreover, HPLC analysis showed that 50B6I6ΔphaEC synthesized lycopene with a production level of 119.25 ± 0.55 mg/g DCW, which was 73.0% higher than that of 50B6I6. Meanwhile, lycopene titer of 50B6I6ΔphaEC increased to 429.41 ± 5.81 mg/L, which was 21% enhancement compared to 50B6I6. This result indicated that the disruption of PHBV biosynthesis could enhance the acetyl-CoA flux to lycopene biosynthesis.

**Table 2:** PHBV content and lycopene production in 50B6I6 and 50B6I6ΔphaEC.

Strains	PHBV %	Lycopene	
		Titer (mg/L)	Production (mg/g)
50B6I6	62.84 ± 1.11	353.06 ± 2.39	68.95 ± 1.19
50B6I6ΔphaEC	0	429.41 ± 5.81	119.25 ± 0.55

### Effect of Auxotrophy on Lycopene Production

**FIGURE 6:**
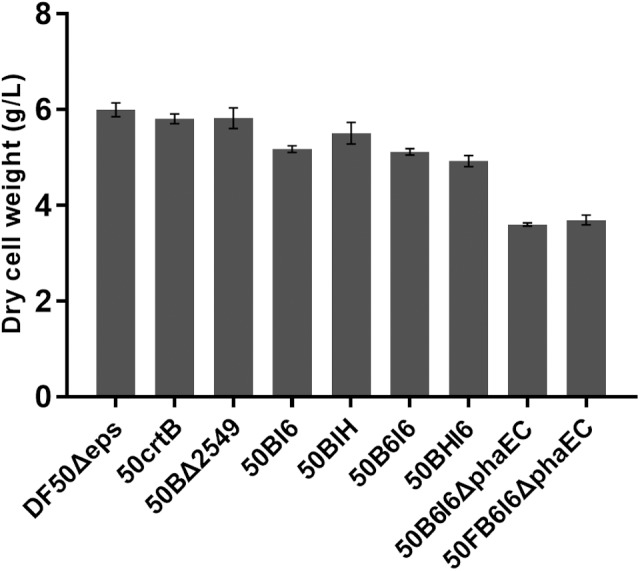
Dry cell weights of engineered *H. mediterranei* strains.

In this work, all the genetic manipulation was based on the *pyrF*-deleted strain DF50Δeps (uracil auxotrophic mutant). Uracil addition was required to culture these engineered strains in MG medium. However, this approach was not suitable for high-cell density fermentation and increased the production cost. To address this issue, we restored the functional expression of *pyrF* in 50B6I6ΔphaEC. A linear DNA fragment containing *pyrF* and homologous arms, 500 bp in up-stream or down-stream of *pyrF*, was amplified by PCR using the genomic DNA of *H. mediterranei* as a template and transferred into the 50B6I6ΔphaEC strain. The screening process was carried out using AS-168SY medium, in which the negative colonies could not grow. We obtained the correct *pyrF* complementary strain 50FB6I6ΔphaEC and it gave a lycopene yield of 107.37 ± 2.37 mg/g DCW (396.70 ± 13.39 mg/L), while there was no difference in biomass between 50B6I6ΔphaEC and 50FB6I6ΔphaEC (Figure 6). Although the complementation of *pyrF* did not increase the biomass and led to a little decrease in lycopene content, 50FB6I6ΔphaEC could be cultured in MG medium without uracil, which was more feasible for industrial application.

## Discussion

Regarding to food safety issues, lycopene from natural source, such as watermelon, gac fruit, tomato, and so on (Perkins-Veazie and Davis, 2004; Viuda-Martos et al., 2014; Lv et al., 2015; Papaioannou et al., 2016; Wimalasiri et al., 2017), is superior to that from chemical synthesis. Among these fruits or vegetables, tomato is a major source of lycopene, but its total lycopene content is too low to meet the market requirements. Multiple strategies of engineering the carotenoid synthesis pathway in tomato fruit to improve its lycopene content were adopted in several studies. Fraser et al. (2002) introduced an additional *crtB* from *Erwinia uredovora* into tomato in a fruit-specific manner and obtained a 1.8-fold increase of lycopene content. Enfissi et al. (2005) got a 1.6-fold increase of lycopene content by overexpression of a bacterial 1-deoxy-D-xylulose-5-phosphate synthase (DXS) encoding gene. Namitha and Negi (2018) introduced a bacterial *crtY* gene from *Pantoea agglomerans* into tomato fruit to enhance lycopene production by 2.1-fold. In addition, other strategies were also used to alter carotenoid content in tomato fruit. Overexpression of blue light photoreceptor, cryptochrome 2, resulted in a 1.7-fold increase of lycopene content in tomato fruit (Giliberto et al., 2005). Importing the pepper fibrillin gene into tomato fruit led to a 118% increase in lycopene level (Simkin et al., 2007). Additionally, Mehta et al. (2002) demonstrated that higher level of polyamines in tomato fruit by fruit-specific overexpression of a yeast *S*-adenosylmethionine decarboxylase gene (*ySAMdc*) enhanced the lycopene content by 2 ∼ 3 folds in tomato fruit. In the same way, Neily et al. (2011) overexpressed the spermidine synthase gene in tomato and also got an unexpected increase of 1.3 ∼ 2.2 folds in lycopene prodcution. The increased polyamines were revealed to affect multiple cell pathways and broad gene expression levels, thereby enhancing lycopene accumulation (Mattoo et al., 2006, 2007; Kolotilin, 2008; Kolotilin et al., 2011; Neily et al., 2011; Guo et al., 2018). However, these transgenic plants are still far from large-scale industrial application for lycopene production (Table 3). Lycopene production by microbial fermentation is an attractive alternative to use of plants. Moreover, the strains also need to be engineered for improving its production to make the fermentation process more cost competitive. In engineered *E. coli*, the highest lycopene production of 448 mg/g DCW was obtained by employing a new combinatorial multi-gene pathway assembly scheme (Coussement et al., 2017). In yeast, through engineering host and pathway, the highest lycopene yield, 55.56 mg/g DCW was achieved in 5-L bioreactors (Chen et al., 2016). In *B. trispora*, a lycopene production of 103.58 mg/g DCW was realized by the modification of the bifunctional gene, *carRA*, combined with addition of tripropylamine (Wang et al., 2017). In this study, a carotenogenic haloarchaea, *H. mediterranei* was chosen as a novel chassis cell for lycopene overproduction, due to its several superior features, such as high salt tolerance capability, easy lysis, etc. These can contribute to reduce the energy cost brought by strict sterilization and to simplify the process for lycopene extraction. We adopted multiple strategies to engineer *H. mediterranei* for lycopene production enhancement and finally our best strain gave a lycopene yield of 119.25 mg/g DCW, which was even higher than the yields of most well-studied strains (Table 3). Although engineered *E. coli* produced the highest level of lycopene, it is controversial to use it for lycopene synthesis, since this strain would release endotoxin (Ray and Raetz, 1987). In contrast, haloarchaea has an extremely low endotoxin level because of its special structure of cell envelope (Xue et al., 2018). Thus, *H. mediterranei* is a promising microbial host for lycopene biosynthesis.

**Table 3:** Summary of lycopene production in transgenic plant and engineered microorganisms.

Organism	Strategies	Culture condition	Lycopene production	Reference
**Plant**					
Tomato	Engineering carotenoids synthesis pathway	Grown in the glasshouse	5.22 mg/g DCW	Fraser et al., 2002
Tomato		Grown in the glasshouse	6.7 mg/g DCW	Enfissi et al., 2005
Tomato		Grown in green house	∼0.11 mg/g DCW	Namitha and Negi, 2018
Tomato	Manipulation of the blue light photoreceptor cryptochrome 2	Grown in green house	1.35 mg/g DCW	Giliberto et al., 2005
Tomato	Importing the pepper fibrillin gene in tomato	Grown in green house	∼0.48 mg/g FW^∗^	Simkin et al., 2007
Tomato	Enhancement of polyamine accumulation	Grown in green house	∼0.11 mg/g FW^∗^	Mehta et al., 2002
Tomato		Grown in green house	1.72 mg/g DCW	Neily et al., 2011
**Microbe**				
50B6I6ΔphaEC	Pathway engineering	Shake flask fermentation	119.25 mg/g DCW	This study
*E. coli*		Microtiter plate fermentation	448 mg/g DCW	Coussement et al., 2017
*E. coli*	Chromosomal evolution	Shake flask fermentation	33.4 mg/g DCW	Chen et al., 2013
*E. coli*	Pathway balancing	Fed-batch fermentation	43.7 mg/g DCW	Zhu et al., 2015
*E. coli*	Pathway engineering combined with NADPH and ATP balancing	Fed-batch fermentation	50.6 mg/g DCW	Tao et al., 2014)
*E. coli*	Plasmid based overexpression of carotenoids synthesis genes	Shake flask fermentation	67 mg/g DCW	Xu et al., 2018
*S. cerevisiae*	Directed evolution and metabolic engineering	Fed-batch fermentation	24.41 mg/g DCW	Xie et al., 2015a
*S. cerevisiae*	Pathway engineering	Fed-batch fermentation	55.56 mg/g DCW	Chen et al., 2016
*Yarrowia lipolytica*		Fed-batch fermentation	21.1 mg/g DCW	Schwartz et al., 2017
*B. trispora*	Genetically manipulated the bifunctional protein gene, *carRA*	Shake flask fermentation	103.58 mg/g DCW	Wang et al., 2017

*^∗^FW, fresh weight.*

However, in optimal culture conditions for growth, *H. mediterranei*, is less pigmented and no lycopene can be detected (Figure 3B). This might be due to the presence of a rate-limiting step involved in lycopene synthesis. Then we investigated the overexpression of the possible rate-limiting enzyme encoding genes in the MVA and lycopene synthesis pathway and found that only *crtB* overexpression resulted in significant lycopene accumulation. This result suggested that the MVA pathway in *H. mediterranei* is efficient to produce essential precursors of IPP and DMAPP for lycopene synthesis. Thus, in the next step, the first strategy we adopted to improve lycopene production was reinforcing lycopene synthesis pathway. First, we eliminated the rate-limiting step by inserting a strong promoter in the chromosome ahead of *crtB* and realized a lycopene production of 6.05 mg/g DCW. Then to avoid the limitation of final target products, brought by insufficient ability of downstream pathway (Leonard et al., 2010), we imported heterologous *crtI* and enhanced the lycopene production to 56.46 mg/g DCW. Next, we integrated heterologous haloarchaeal *crtB-crtI cassettes* into the chromosome and got a lycopene yield of 68.95 mg/g DCW. Similarly, Xie et al. (2015a) adjusted the copy number of *crt* genes to get more than 80% increase of lycopene production in *Saccharomyces cerevisiae*. They reported that multiple copies of *crt* genes led to an about 13% decrease in biomass (Xie et al., 2015a). Similar results were also obtained in the present study (Figure 6). The dry cell weight of the engineered strains 50BI6, 50BIH, 50B6I6, and 50BHI6 showed a decrease of about 10%. This might be due to the metabolic burden brought by the overexpression of *crt* genes. However, the significant increase of lycopene titer overweighed the slight biomass decrease in these engineered strains.

The second strategy we used is disruption of the lycopene flux to other carotenoids or deletion of the competing pathways sharing common precursors with the lycopene synthetic pathway. Wang et al. (2016) inhibited the activity of lycopene cyclase, the enzyme responsible for conversion of lycopene to β-carotene, and increased the lycopene content by 90.1%. In *E. coli*, the knockout of *gdhA*, *accE*, and *fdhF* gave a 37% increase in lycopene content (Alper et al., 2005). In this work, we knocked out the gene *lyeJ* to block the bacterioruberin biosynthesis and thus improved the lycopene purity and got a 6.5-fold increase of lycopene production. MVA pathway commences with acetyl-CoA, which is also the important precursor for PHBV synthesis in *H. mediterranei*. Removing the competing pathways for lycopene synthesis can theoretically facilitate lycopene accumulation. So we disrupted the PHBV synthesis in *H. mediterranei*, by deleting the key genes *phaE* and *phaC*. As expected, the engineered strain 50B6I6ΔphaEC did not synthesis PHBV and showed an increase of lycopene titer as expected. The loss of PHBV caused a decrease in dry cell weight by 42% (Figure 6). On the other hand, the lycopene production was sharply enhanced by 73%. This indicated that more acetyl-CoA could flux to lycopene synthesis *via* MVA pathway with the disruption of PHBV synthesis.

High biomass is necessary to achieve a high yield of lycopene. However, the presence of auxotrophies can cause an organism to grow more slowly than the equivalent prototroph (Pronk, 2002). Furthermore, it is not feasible for high-cell density fermentation and practical application because of the requirement of additional uracil. In *Y. lipolytica*, the alleviation of both *leu2* and *ura3* auxotrophies gave a 1.9-fold enhancement in lycopene titer (Schwartz et al., 2017). In this work, we complemented the *pyrF* auxotrophy in strain 50B6I6ΔphaEC. The engineered strain 50FB6I6ΔphaEC showed no difference in dry cell weight compared with 50B6I6ΔphaEC (Figure 6), but gave a little decrease in lycopene titer and production. The low lycopene accumulation after the complementation of *pyrF* gene might be ascribed to the fact that the utilization efficiency of the uracil synthesized *in vivo* was lower than that of the uracil added for cell growth. However, no need for uracil overweighed the slight decrease of lycopene production as for developing a cost-effective industrial strain.

This work reveals that *H. mediterranei* possesses a great potential for lycopene biosynthesis and much more efforts are needed to further increase its lycopene production. The reduction of FPP flux to the squalene biosynthetic pathway (a competing pathway for carotenoid synthesis) is expected to further increase lycopene yield. This strategy has been used to improve lycopene production in yeast (Xie et al., 2015b). Besides, modulation of the NADPH and ATP levels is another alternative approach for further enhancing lycopene synthesis in *H. mediterranei*. Additionally, optimization of culture conditions and fed-batch fermentation might also be adopted for maximizing lycopene production in *H. mediterranei*.

## Conclusion

In this work, we engineered a haloarchaeon, *H. mediterranei*, as a novel host for lycopene overproduction by adopting multiple strategies. Introducing a constitutive promoter enhanced the expression level of the rate-limiting enzyme encoding gene, *crtB*, and disrupting the bacterioruberin synthesis significantly increased the lycopene production and purity. Importing different heterologous *crt* expression cassettes were also an effective method for improving lycopene production. Further blocking PHBV synthesis to direct more acetyl-CoA flux into carotenoid synthesis showed a dramatic increase in lycopene production, up to 119.25 ± 0.55 mg/g DCW, in shake flask fermentation. Complementation of *pyrF* in the engineered strain 50B6I6ΔphaEC had no increase in both dry cell weight and lycopene production, but it could grow without addition of uracil and thus is more suitable for industrial application. The engineering pathway that we developed in this study shows the potential for high-level production of lycopene and offers biological insights into carotenoid production in haloarchaea.

## Author Contributions

Z-QZ, JH, and HX conceived the project, analyzed the data, and drafted the manuscript. Z-QZ and JZ performed the study. QX, D-HZ, and JH critically revised the manuscript. All authors read and approved the final manuscript.

## Conflict of Interest Statement

The authors declare that the research was conducted in the absence of any commercial or financial relationships that could be construed as a potential conflict of interest.

## Footnotes

1 http://www.uniprot.org/

2 http://www.cbs.dtu.dk/services/TMHMM/

## References

[B1] Alper H. Jin Y.-S. Moxley J. F. Stephanopoulos G. (2005). Identifying gene targets for the metabolic engineering of lycopene biosynthesis in *Escherichia coli*. *Metab. Eng.* 7 155–164. 10.1016/j.ymben.2004.12.003 15885614

[B2] Alper H. Stephanopoulos G. (2008). Uncovering the gene knockout landscape for improved lycopene production in *E. coli*. *Appl. Microbiol. Biotechnol.* 78 801–810. 10.1007/s00253-008-1373-x 18239914

[B3] Altschul S. F. Gish W. Miller W. Myers E. W. Lipman D. J. (1990). Basic local alignment search tool. *J. Mol. Biol.* 215 403–410. 10.1006/jmbi.1990.9999 2231712

[B4] Anthony J. R. Anthony L. C. Nowroozi F. Kwon G. Newman J. D. Keasling J. D. (2009). Optimization of the mevalonate-based isoprenoid biosynthetic pathway in *Escherichia coli* for production of the anti-malarial drug precursor amorpha-4,11-diene. *Metab. Eng.* 11 13–19. 10.1016/j.ymben.2008.07.007 18775787

[B5] Antón J. Meseguer I. Rodríguezvalera F. (1988). Production of an extracellular polysaccharide by *Haloferax mediterranei*. *Appl. Environ. Microbiol.* 54 2381–2386. 16347749 10.1128/aem.54.10.2381-2386.1988 PMC204266

[B6] Azami Y. Hattori A. Nishimura H. Kawaide H. Yoshimura T. Hemmi H. (2014). (R)-mevalonate 3-phosphate is an intermediate of the mevalonate pathway in *Thermoplasma acidophilum*. *J. Biol. Chem.* 289 15957–15967. 10.1074/jbc.M114.562686 24755225 PMC4047369

[B7] Berthelot K. Estevez Y. Deffieux A. Peruch F. (2012). Isopentenyl diphosphate isomerase: a checkpoint to isoprenoid biosynthesis. *Biochimie* 94 1621–1634. 10.1016/j.biochi.2012.03.021 22503704

[B8] Bhattacharyya A. Saha J. Haldar S. Bhowmic A. Mukhopadhyay U. K. Mukherjee J. (2014). Production of poly-3-(hydroxybutyrate-co-hydroxyvalerate) by *Haloferax mediterranei* using rice-based ethanol stillage with simultaneous recovery and re-use of medium salts. *Extremophiles* 18 463–470. 10.1007/s00792-013-0622-9 24442255

[B9] Bischoff K. M. Rodwell V. W. (1996). 3-Hydroxy-3-methylglutaryl-coenzyme a reductase from *Haloferax volcanii*: purification, characterization, and expression in *Escherichia coli*. *J. Bacteriol.* 178 19–23. 10.1128/jb.178.1.19-23.1996 8550415 PMC177616

[B10] Cai S. Cai L. Liu H. Liu X. Han J. Zhou J. (2012). Identification of the haloarchaeal phasin (PhaP) that functions in polyhydroxyalkanoate accumulation and granule formation in *Haloferax mediterranei*. *Appl. Environ. Microbiol.* 78 1946–1952. 10.1128/AEM.07114-11 22247127 PMC3298179

[B11] Cai S. F. Cai L. Zhao D. H. Liu G. M. Han J. Zhou J. (2015). A novel DNA-binding protein, PhaR, plays a central role in the regulation of polyhydroxyalkanoate accumulation and granule formation in the haloarchaeon *Haloferax mediterranei*. *Appl. Environ. Microbiol.* 81 373–385. 10.1128/aem.02878-14 25344243 PMC4272714

[B12] Chamovitz D. Misawa N. Sandmann G. Hirschberg J. (1992). Molecular cloning and expression in *Escherichia coli* of a cyanobacterial gene coding for phytoene synthase, a carotenoid biosynthesis enzyme. *FEBS Lett.* 296 305–310. 10.1016/0014-5793(92)80310-D 1537409

[B13] Chen C. W. Hsu S. H. Lin M. T. Hsu Y. H. (2015). Mass production of C-50 carotenoids by *Haloferax mediterranei* in using extruded rice bran and starch under optimal conductivity of brined medium. *Bioprocess. Biosyst. Eng.* 38 2361–2367. 10.1007/s00449-015-1471-y 26373421

[B14] Chen Y. Xiao W. H. Wang Y. Liu H. Li X. Yuan Y. J. (2016). Lycopene overproduction in *Saccharomyces cerevisiae* through combining pathway engineering with host engineering. *Microb. Cell Fact.* 15:113. 10.1186/s12934-016-0509-4 27329233 PMC4915043

[B15] Chen Y. Y. Shen H. J. Cui Y. Y. Chen S. G. Weng Z. M. Zhao M. (2013). Chromosomal evolution of *Escherichia coli* for the efficient production of lycopene. *BMC Biotechnol.* 13:6. 10.1186/1472-6750-13-6 23356604 PMC3626847

[B16] Cline S. W. Lam W. L. Charlebois R. L. Schalkwyk L. C. Doolittle W. F. (1989). Transformation methods for halophilic archaebacteria. *Can. J. Microbiol.* 35 148–152. 10.1139/m89-022 2497937

[B17] Coussement P. Bauwens D. Maertens J. De Mey M. (2017). Direct combinatorial pathway optimization. *ACS Synth. Biol.* 6 224–232. 10.1021/acssynbio.6b00122 27672702

[B18] de Lourdes Moreno M. Sanchez-Porro C. Garcia M. T. Mellado E. (2012). Carotenoids’ production from halophilic bacteria. *Methods Mol. Biol.* 892 207–217. 10.1007/978-1-61779-879-5-12 22623305

[B19] Don T. M. Chen C. W. Chan T. H. (2006). Preparation and characterization of poly(hydroxyalkanoate) from the fermentation of *Haloferax mediterranei. Sci. Polym. Ed.* 17 1425–1438. 10.1163/156856206778937208 17260512

[B20] Enfissi E. M. A. Fraser P. D. Lois L.-M. Boronat A. Schuch W. Bramley P. M. (2005). Metabolic engineering of the mevalonate and non-mevalonate isopentenyl diphosphate-forming pathways for the production of health-promoting isoprenoids in tomato. *Plant Biotechnol. J.* 3 17–27. 10.1111/j.1467-7652.2004.00091.x 17168896

[B21] Fang C. J. Ku K. L. Lee M. H. Su N. W. (2010). Influence of nutritive factors on C-50 carotenoids production by *Haloferax mediterranei* ATCC 33500 with two-stage cultivation. *Bioresour. Technol.* 101 6487–6493. 10.1016/j.biortech.2010.03.044 20362434

[B22] Fraser P. D. Misawa N. Linden H. Yamano S. Kobayashi K. Sandmann G. (1992). Expression in *Escherichia coli*, purification, and reactivation of the recombinant *Erwinia uredovora* phytoene desaturase. *J. Biol. Chem.* 267 19891–19895. 1400305

[B23] Fraser P. D. Romer S. Shipton C. A. Mills P. B. Kiano J. W. Misawa N. (2002). Evaluation of transgenic tomato plants expressing an additional phytoene synthase in a fruit-specific manner. *Proc. Natl. Acad. Sci. U.S.A.* 99 1092–1097. 10.1073/pnas.241374598 11805345 PMC117435

[B24] Gajowik A. Dobrzynska M. M. (2014). Lycopene - antioxidant with radioprotective and anticancer properties. A review. *Rocz. Panstw. Zakl. Hig.* 65 263–271. 25526570

[B25] Giliberto L. Perrotta G. Pallara P. Weller J. L. Fraser P. D. Bramley P. M. (2005). Manipulation of the blue light photoreceptor cryptochrome 2 in tomato affects vegetative development, flowering time, and fruit antioxidant content. *Plant Physiol.* 137 199–208. 10.1104/pp.104.051987 15618424 PMC548851

[B26] Goldstein J. L. Brown M. S. (1990). Regulation of the mevalonate pathway. *Nature* 343 425–430. 10.1038/343425a0 1967820

[B27] Guo J. Wang S. Yu X. Dong R. Li Y. Mei X. (2018). Polyamines regulate strawberry fruit ripening by abscisic acid, auxin, and ethylene. *Plant Physiol.* 177 339–351. 10.1104/pp.18.00245 29523717 PMC5933135

[B28] Han J. Hou J. Zhang F. Ai G. Li M. Cai S. (2013). Multiple propionyl coenzyme A-supplying pathways for production of the bioplastic poly(3-hydroxybutyrate-co-3-hydroxyvalerate) in *Haloferax mediterranei*. *Appl. Environ. Microbiol.* 79 2922–2931. 10.1128/aem.03915-12 23435886 PMC3623125

[B29] Han J. Lu Q. Zhou L. Zhou J. Xiang H. (2007). Molecular characterization of the phaEC(Hm) genes, required for biosynthesis of poly(3-hydroxybutyrate) in the extremely halophilic archaeon *Haloarcula marismortui*. *Appl. Environ. Microbiol.* 73 6058–6065. 10.1128/AEM.00953-07 17675423 PMC2075026

[B30] Han J. Wu L. P. Liu X. B. Hou J. Zhao L. L. Chen J. Y. (2017). Biodegradation and biocompatibility of haloarchaea-produced poly(3-hydroxybutyrate-co-3-hydroxyvalerate) copolymers. *Biomaterials* 139 172–186. 10.1016/j.biomaterials.2017.06.006 28618347

[B31] Han J. Zhang F. Hou J. Liu X. Li M. Liu H. (2012). Complete genome sequence of the metabolically versatile halophilic archaeon *Haloferax mediterranei*, a poly(3-hydroxybutyrate-co-3-hydroxyvalerate) producer. *J. Bacteriol.* 194 4463–4464. 10.1128/JB.00880-12 22843593 PMC3416209

[B32] Hemmi H. Ohnuma S. Nagaoka K. Nishino T. (1998). Identification of genes affecting lycopene formation in *Escherichia coli* transformed with carotenoid biosynthetic genes: candidates for early genes in isoprenoid biosynthesis. *J. Biochem.* 123 1088–1096. 10.1093/oxfordjournals.jbchem.a022047 9603997

[B33] Hosfield D. J. Zhang Y. Dougan D. R. Broun A. Tari L. W. Swanson R. V. (2004). Structural basis for bisphosphonate-mediated inhibition of isoprenoid biosynthesis. *J. Biol. Chem.* 279 8526–8529. 10.1074/jbc.C300511200 14672944

[B34] Kang M. J. Lee Y. M. Yoon S. H. Kim J. H. Ock S. W. Jung K. H. (2005). Identification of genes affecting lycopene accumulation in *Escherichia coli* using a shot-gun method. *Biotechnol. Bioeng.* 91 636–642. 10.1002/bit.20539 15898075

[B35] Kolotilin I. (2008). *Assessment of Transgenic Approaches to Increase Lycopene Content in Ripe Tomato Fruits.* Ph.D. thesis. Hebrew University, Jerusalem.

[B36] Kolotilin I. Koltai H. Bar-Or C. Chen L. Nahon S. Shlomo H. (2011). Expressing yeast *SAMdc* gene confers broad changes in gene expression and alters fatty acid composition in tomato fruit. *Physiol. Plant* 142 211–223. 10.1111/j.1399-3054.2011.01458.x 21338368

[B37] Krogh A. Larsson B. von Heijne G. Sonnhammer E. L. L. (2001). Predicting transmembrane protein topology with a hidden Markov model: application to complete genomes. *J. Mol. Biol.* 305 567–580. 10.1006/jmbi.2000.4315 11152613

[B38] Leonard E. Ajikumar P. K. Thayer K. Xiao W.-H. Mo J. D. Tidor B. (2010). Combining metabolic and protein engineering of a terpenoid biosynthetic pathway for overproduction and selectivity control. *Proc. Natl. Acad. Sci. U.S.A.* 107 13654–13659. 10.1073/pnas.1006138107 20643967 PMC2922259

[B39] Liu H. L. Han J. Liu X. Q. Zhou J. Xiang H. (2011). Development of *pyrF*-based gene knockout systems for genome-wide manipulation of the archaea *Haloferax mediterranei* and *Haloarcula hispanica*. *J. Genet. Genomics* 38 261–269. 10.1016/j.jgg.2011.05.003 21703550

[B40] Liu X.-J. Liu R.-S. Li H.-M. Tang Y.-J. (2012). Lycopene production from synthetic medium by *Blakeslea trispora* NRRL 2895 (+) and 2896 (-) in a stirred-tank fermenter. *Bioprocess. Biosyst. Eng.* 35 739–749. 10.1007/s00449-011-0654-4 22105931

[B41] Lombard J. Moreira D. (2011). Origins and early evolution of the mevalonate pathway of isoprenoid biosynthesis in the three domains of life. *Mol. Biol. Evol.* 28 87–99. 10.1093/molbev/msq177 20651049

[B42] Lu Q. Han J. Zhou L. Coker J. A. DasSarma P. DasSarma S. (2008a). Dissection of the regulatory mechanism of a heat-shock responsive promoter in haloarchaea: a new paradigm for general transcription factor directed archaeal gene regulation. *Nucleic Acids Res.* 36 3031–3042. 10.1093/nar/gkn152 18390887 PMC2396416

[B43] Lu Q. Han J. Zhou L. Zhou J. Xiang H. (2008b). Genetic and biochemical characterization of the poly(3-hydroxybutyrate-co-3-hydroxyvalerate) synthase in *Haloferax mediterranei*. *J. Bacteriol.* 190 4173–4180. 10.1128/jb.00134-08 18408025 PMC2446746

[B44] Lv P. Li N. Liu H. Gu H. Zhao W.-E. (2015). Changes in carotenoid profiles and in the expression pattern of the genes in carotenoid metabolisms during fruit development and ripening in four watermelon cultivars. *Food Chem.* 174 52–59. 10.1016/j.foodchem.2014.11.022 25529651

[B45] Mattoo A. K. Chung S. H. Goyal R. K. Fatima T. Solomos T. Srivastava A. (2007). Overaccumulation of higher polyamines in ripening transgenic tomato fruit revives metabolic memory, upregulates anabolism-related genes, and positively impacts nutritional quality. *J. AOAC Int.* 90 1456–1464. 17955994

[B46] Mattoo A. K. Sobolev A. P. Neelam A. Goyal R. K. Handa A. K. Segre A. L. (2006). Nuclear magnetic resonance spectroscopy-based metabolite profiling of transgenic tomato fruit engineered to accumulate spermidine and spermine reveals enhanced anabolic and nitrogen-carbon interactions. *Plant Physiol.* 142 1759–1770. 10.1104/pp.106.084400 17041034 PMC1676058

[B47] Mehta R. A. Cassol T. Li N. Ali N. Handa A. K. Mattoo A. K. (2002). Engineered polyamine accumulation in tomato enhances phytonutrient content, juice quality, and vine life. *Nat. Biotechnol.* 20 613–618. 10.1038/nbt0602-613 12042867

[B48] Namitha K. K. Negi P. S. (2018). Transformation of tomato cv. arka ahuti (*Solanum lycopersicum* L.) with phytoene desaturase (CrtI) and lycopene β-cyclase (CrtY) genes increases carotenoid content and antioxidant potential. *J. Plant Biochem. Biotechnol.* 27 68–77. 10.1007/s13562-017-0417-7

[B49] Naziri D. Hamidi M. Hassanzadeh S. Tarhriz V. Maleki Zanjani B. Nazemyieh H. (2014). Analysis of carotenoid production by *Halorubrum* sp. TBZ126; an extremely halophilic archeon from Urmia Lake. *Adv. Pharm. Bull.* 4 61–67. 10.5681/apb.2014.010 24409411 PMC3885371

[B50] Neily M. H. Matsukura C. Maucourt M. Bernillon S. Deborde C. Moing A. (2011). Enhanced polyamine accumulation alters carotenoid metabolism at the transcriptional level in tomato fruit over-expressing spermidine synthase. *J. Plant Physiol.* 168 242–252. 10.1016/j.jplph.2010.07.003 20708298

[B51] Oren A. Hallsworth J. E. (2014). Microbial weeds in hypersaline habitats: the enigma of the weed-like *Haloferax mediterranei*. *FEMS Microbiol. Lett.* 359 134–142. 10.1111/1574-6968.12571 25132231

[B52] Palmer B. R. Marinus M. G. (1994). The *dam* and *dcm* strains of *Escherichia coli* - a review. *Gene* 143 1–12. 10.1016/0378-1119(94)90597-5 8200522

[B53] Papaioannou E. H. Liakopoulou-Kyriakides M. Karabelas A. J. (2016). Natural origin lycopene and its “Green” downstream processing. *Crit. Rev. Food Sci. Nutr.* 56 686–709. 10.1080/10408398.2013.817381 25671774

[B54] Peck R. F. Echavarri-Erasun C. Johnson E. A. Ng W. V. Kennedy S. P. Hood L. (2001). *Brp* and *blh* are required for synthesis of the retinal cofactor of bacteriorhodopsin in *Halobacterium salinarum*. *J. Biol. Chem.* 276 5739–5744. 10.1074/jbc.M009492200 11092896

[B55] Peck R. F. Johnson E. A. Krebs M. P. (2002). Identification of a lycopene beta-cyclase required for bacteriorhodopsin biogenesis in the archaeon *Halobacterium salinarum*. *J. Bacteriol.* 184 2889–2897. 10.1128/jb.184.11.2889-2897.2002 12003928 PMC135044

[B56] Peck R. F. Plesa A. M. Graham S. M. Angelini D. R. Shaw E. L. (2017). Opsin-mediated inhibition of bacterioruberin synthesis in halophilic archaea. *J. Bacteriol.* 199:e00303-17. 10.1128/jb.00303-17 28784816 PMC5626960

[B57] Perkins-Veazie P. Davis A. R. (2004). In search of high lycopene watermelon. *Cucurbit Genet. Coop. Rep.* 27 51–53.

[B58] Pronk J. T. (2002). Auxotrophic yeast strains in fundamental and applied research. *Appl. Environ. Microbiol.* 68 2095–2100. 10.1128/AEM.68.5.2095-2100.2002 11976076 PMC127579

[B59] Ray B. L. Raetz C. R. (1987). The biosynthesis of gram-negative endotoxin. A novel kinase in *Escherichia coli* membranes that incorporates the 4′-phosphate of lipid A. *J. Biol. Chem.* 262 1122–1128. 3027079

[B60] Rodrigo-Banos M. Garbayo I. Vilchez C. Bonete M. J. Martinez-Espinosa R. M. (2015). Carotenoids from haloarchaea and their potential in biotechnology. *Mar. Drugs* 13 5508–5532. 10.3390/md13095508 26308012 PMC4584337

[B61] Sambrook H. C. (1989). *Molecular Cloning : A Laboratory Manual.* Cold Spring Harbor, NY: Cold Spring Harbor Laboratory.

[B62] Schwartz C. Frogue K. Misa J. Wheeldon I. (2017). Host and pathway engineering for enhanced lycopene biosynthesis in *Yarrowia lipolytica*. *Front. Microbiol.* 8:11. 10.3389/fmicb.2017.02233 29276501 PMC5727423

[B63] Sies H. Stahl W. (1998). Lycopene: antioxidant and biological effects and its bioavailability in the human. *Proc. Soc. Exp. Biol. Med.* 218 121–124. 10.3181/00379727-218-44285a 9605209

[B64] Simkin A. J. Gaffé J. Alcaraz J.-P. Carde J.-P. Bramley P. M. Fraser P. D. (2007). Fibrillin influence on plastid ultrastructure and pigment content in tomato fruit. *Phytochemistry* 68 1545–1556. 10.1016/j.phytochem.2007.03.014 17466343

[B65] Singh A. Singh A. K. (2017). Haloarchaea: worth exploring for their biotechnological potential. *Biotechnol. Lett.* 39 1793–1800. 10.1007/s10529-017-2434-y 28900776

[B66] Steussy C. N. Vartia A. A. Burgner J. W. II Sutherlin A. Rodwell V. W. Stauffacher C. V. (2005). X-ray crystal structures of HMG-CoA synthase from *Enterococcus faecalis* and a complex with its second substrate/inhibitor acetoacetyl-CoA. *Biochemistry* 44 14256–14267. 10.1021/bi051487x 16245942

[B67] Strand A. Shivaji S. Liaaen-Jensen S. (1997). Bacterial carotenoids 55. C50-carotenoids 25.† revised structures of carotenoids associated with membranes in psychrotrophic Micrococcus roseus. *Biochem. Syst. Ecol.* 25 547–552. 10.1016/S0305-1978(97)00039-2

[B68] Su A. Chi S. Li Y. Tan S. Qiang S. Chen Z. (2018). Metabolic redesign of *Rhodobacter sphaeroides* for lycopene production. *J. Agric. Food Chem.* 66 5879–5885. 10.1021/acs.jafc.8b00855 29806774

[B69] Tao S. Miao L. Li Q. Dai G. Lu F. Tao L. (2014). Production of lycopene by metabolically-engineered *Escherichia coli*. *Biotechnol. Lett.* 36 1515–1522. 10.1007/s10529-014-1543-0 24806808

[B70] VanNice J. C. Skaff D. A. Keightley A. Addo J. K. Wyckoff G. J. Miziorko H. M. (2014). Identification in *Haloferax volcanii* of phosphomevalonate decarboxylase and isopentenyl phosphate kinase as catalysts of the terminal enzyme reactions in an archaeal alternate mevalonate pathway. *J. Bacteriol.* 196 1055–1063. 10.1128/jb.01230-13 24375100 PMC3957691

[B71] VanNice J. C. Skaff D. A. Wyckoff G. J. Miziorko H. M. (2013). Expression in *Haloferax volcanii* of 3-hydroxy-3-methylglutaryl coenzyme A synthase facilitates isolation and characterization of the active form of a key enzyme required for polyisoprenoid cell membrane biosynthesis in halophilic archaea. *J. Bacteriol.* 195 3854–3862. 10.1128/jb.00485-13 23794621 PMC3754609

[B72] Viuda-Martos M. Sanchez-Zapata E. Sayas-Barbera E. Sendra E. Perez-Alvarez J. A. Fernandez-Lopez J. (2014). Tomato and tomato byproducts. Human health benefits of lycopene and its application to meat products: a review. *Crit. Rev. Food Sci. Nutr.* 54 1032–1049. 10.1080/10408398.2011.623799 24499120

[B73] Wang Y. Chen X. Hong X. Du S. Liu C. Gong W. (2016). Cyclase inhibitor tripropylamine significantly enhanced lycopene accumulation in *Blakeslea trispora*. *J. Biosci. Bioeng.* 122 570–576. 10.1016/j.jbiosc.2016.05.001 27238833

[B74] Wang Y. L. Pang J. Zheng Y. M. Jiang P. P. Gong W. F. Chen X. W. (2017). Genetic manipulation of the bifunctional gene, carRA, to enhance lycopene content in *Blakeslea trispora*. *Biochem. Eng. J.* 119 27–33. 10.1016/j.bej.2016.12.011

[B75] Wei Y. Mohsin A. Hong Q. Guo M. Fang H. (2017). Enhanced production of biosynthesized lycopene via heterogenous MVA pathway based on chromosomal multiple position integration strategy plus plasmid systems in *Escherichia coli*. *Bioresour. Technol.* 250 382–389. 10.1016/j.biortech.2017.11.035 29195149

[B76] Wimalasiri D. Brkljaca R. Piva T. J. Urban S. Huynh T. (2017). Comparative analysis of carotenoid content in *Momordica cochinchinensis* (Cucurbitaceae) collected from Australia, Thailand and Vietnam. *J. Food Sci. Technol.* 54 2814–2824. 10.1007/s13197-017-2719-0 28928521 PMC5583111

[B77] Wu T. Ye L. Zhao D. Li S. Li Q. Zhang B. (2018). Engineering membrane morphology and manipulating synthesis for increased lycopene accumulation in *Escherichia coli* cell factories. *3 Biotech* 8:269. 10.1007/s13205-018-1298-8 29868307 PMC5970105

[B78] Xie W. Lv X. Ye L. Zhou P. Yu H. (2015a). Construction of lycopene-overproducing *Saccharomyces cerevisiae* by combining directed evolution and metabolic engineering. *Metab. Eng.* 30 69–78. 10.1016/j.ymben.2015.04.009 25959020

[B79] Xie W. Ye L. Lv X. Xu H. Yu H. (2015b). Sequential control of biosynthetic pathways for balanced utilization of metabolic intermediates in *Saccharomyces cerevisiae*. *Metab. Eng.* 28 8–18. 10.1016/j.ymben.2014.11.007 25475893

[B80] Xu J. Xu X. Xu Q. Zhang Z. Jiang L. Huang H. (2018). Efficient production of lycopene by engineered *E. coli* strains harboring different types of plasmids. *Bioprocess. Biosyst. Eng.* 41 489–499. 10.1007/s00449-017-1883-y 29313097

[B81] Xue Q. Liu X. B. Lao Y. H. Wu L. P. Wang D. Zuo Z. Q. (2018). Anti-infective biomaterials with surface-decorated tachyplesin I. *Biomaterials* 178 351–362. 10.1016/j.biomaterials.2018.05.008 29778319

[B82] Zhao D. Cai L. Wu J. Li M. Liu H. Han J. (2013). Improving polyhydroxyalkanoate production by knocking out the genes involved in exopolysaccharide biosynthesis in *Haloferax mediterranei*. *Appl. Microbiol. Biotechnol.* 97 3027–3036. 10.1007/s00253-012-4415-3 23015099

[B83] Zhu F. Lu L. Fu S. Zhong X. Hu M. Deng Z. (2015). Targeted engineering and scale up of lycopene overproduction in *Escherichia coli*. *Process Biochem.* 50 341–346. 10.1016/j.procbio.2014.12.008

