# Supplementary material for: Engineering Haloferax mediterranei as an Efficient Platform for High Level Production of Lycopene
Source: Front Microbiol. 2018 Nov 29;9:2893. doi: 10.3389/fmicb.2018.02893 (PMC6282799; doi:10.3389/fmicb.2018.02893)
Supplement: Supplementary file 1 [file Table_1.DOC]

Supplementary Material

# Engineering *Haloferax mediterranei* as an efficient platform for high level production of lycopene

Zhen-Qiang Zuo^1,2^, Qiong Xue^1,2^, Jian Zhou^1^, Da-He Zhao^1^, Jing Han^1,2*^ and Hua Xiang^1,2*^

*** Correspondence:**Hua Xiang (Tel./fax: +86 10 64807472; E-mail: xiangh@im.ac.cn) and Jing Han (Tel./fax: +86 10 64807475; E-mail: hanjing@im.ac.cn)

Supplementary Table S1. Strains and plasmids used in this study

| **Strains** | **Relevant characteristics** | **Source or reference** |
| --- | --- | --- |
| *E. coli* JM109 | *recA1 supE44 endA1 hsdR17 gyrA96 relA1 thi* | (Sambrook, 1989) |
| *E. coli* JM110 | *dam dcm* mutant of *E. coli* JM109 | (Palmer and Marinus, 1994) |
| DF50Δeps | *pyrF* and *eps* deletion mutant of *H. mediterranei* | (Zhao et al., 2013) |
| 50crtB | P*_phaR_* insertion mutant, upstream of *crtB* in DF50Δeps | This study |
| 50BΔ2549 | *HFX_2549* deletion mutant of 50crtB | This study |
| 50BI6 | *HAH_1058* insertion mutant of 50crtB at *HFX_2549* site | This study |
| 50BIH | *OE_3381R* insertion mutant of 50crtB at *HFX_2549* site | This study |
| 50B6I6 | *HAH_2563* and *HAH_1058* insertion mutant of 50crtB at *HFX_2549* site | This study |
| 50BHI6 | *OE_3093R* and *HAH_1058* insertion mutant of 50crtB at *HFX_2549* site | This study |
| 50B6I6ΔphaEC | *phaEC* deletion mutant of 50B6I6 | This study |
| 50FB6I6ΔphaEC | *pyrF* complementation mutant of 50B6I6ΔphaEC | This study |
| DF50-2550 | DF50Δeps containing pW2550 | This study |
| DF50-2773/4 | DF50Δeps containing pW2773/4 | This study |
| DF50-1486 | DF50Δeps containing pW1486 | This study |
| DF50-2735 | DF50Δeps containing pW2735 | This study |
| DF50-2424 | DF50Δeps containing pW2424 | This study |
| DF50-2547 | DF50Δeps containing pW2547 | This study |
| DF50-2519 | DF50Δeps containing pW2519 | This study |
| DF50-2609 | DF50Δeps containing pW2609 | This study |
|  |  |  |
| **Plasmids** | **Relevant characteristics** | **Source or reference** |
| pWL502 | 7.8-kb; expression vector containing *pyrF* and its native promoter, Ampr | (Cai et al., 2012) |
| pWLR | 7.9-kb; expression vector containing *pyrF* and its native promoter, Ampr and P*_phaR_* | This study |
| pHFX | 4.0-kb; integration vector containing *pyrF* and its native promoter, Ampr | (Liu et al., 2011) |
| pW2550 | 9.4-kb; expression vector pWL502 containing gene *HFX_2550* and P*_phaR_* | This study |
| pW2773-4 | 9.7-kb; expression vector pWL502 containing gene *HFX_2773* and *HFX_2774* and P*_phaR_* | This study |
| pW1486 | 8.9-kb; expression vector pWL502 containing gene *HFX_1486* and P*_phaR_* | This study |
| pW2735 | 9.0-kb; expression vector pWL502 containing gene *HFX_2735* and P*_phaR_* | This study |
| pW2424 | 9.2-kb; expression vector pWL502 containing gene *HFX_2424* and P*_phaR_* | This study |
| pW2547 | 8.8-kb; expression vector pWL502 containing gene *HFX_2547*and P*_phaR_* | This study |
| pW2519 | 8.4-kb; expression vector pWL502 containing gene *HFX_2519* and P*_phaR_* | This study |
| pW2609 | 9.1-kb; expression vector pWL502 containing gene *HFX_2609* and P*_phaR_* | This study |
| pWHA1058 | 9.3-kb; expression vector pWL502 containing gene *HAH_1058* and P*_phaR_* | This study |
| pWHA2563 | 8.7-kb; expression vector pWL502 containing gene *HAH_2563* and P*_phaR_* | This study |
|  |  |  |
| **Plasmids** | **Relevant characteristics** | **Source or reference** |
| pWOE3381 | 9.4-kb; expression vector pWL502 containing gene *OE_3381R* and P*_phaR_* | This study |
| pWOE3093 | 8.8-kb; expression vector pWL502 containing gene *OE_3093R* and P*_phaR_* | This study |
| pHFXB | 5.1-kb; integration vector of pHFX for insertion of P*_phaR_* immediately up-stream of *crtB* | This study |
| pHFX2549K | 5.0-kb; integration vector of pHFX for knocking out *HFX_2549* | This study |
| pHFXPK | 5.1-kb; integration vector of pHFX for knocking out *phaE and phaC* | This study |
| pHI6 | 6.6-kb; integration vector of pHFX for knocking in *HAH_1058* | This study |
| pHIH | 6.7-kb; integration vector of pHFX for knocking in *OE_3381R* | This study |
| pB6I6 | 7.6-kb; integration vector of pHFX for knocking in *HAH_1058* and *HAH_2563* | This study |
| pBHI6 | 7.6-kb; integration vector of pHFX for knocking in *HAH_1058* and *OE_3093R* | This study |

**Supplementary Table S2.** Primers used in this study

Restriction endonuclease sites are underlined.

| **Primers** | **Sequence (5'→3')** | **Usage** |
| --- | --- | --- |
| *phaR*1 | CCATGGATCTAATGGTGTCGAAGGGA | Amplification of promoter P*_phaR_* inserted into pWL502 to construct pWLR |
| *phaR*2 | GGTACCTCTAGAGATATCGGATCCCATCTCCTAACTCGGTGTTG |
| *HFX_2773-4*-F | TTGGATCCGGTACCTGAATGACCGTTTCGAGCGCTCC | Amplification of *HFX_2773* and *HFX_2774* genes inserted into pWLR to construct pW2773/4 |
| *HFX_2773-4*-R | TGTCTAGAGCTCTGAGAGCGACCGATAC |
| *HFX_1486*-F | TTGGATCCGATATCTGAATGAAGGCGACCGCCAAG | Amplification of *HFX_1486* gene inserted into pWLR to construct pW1486 |
| *HFX_1486*-R | TAGGTACCTCTAGACACCTCGGACGCAAGTGA |
| *HFX_2735*-F | TTGGATCCTGAATGAGCCCGGACGCGACGGA | Amplification of *HFX_2735* gene inserted into pWLR to construct pW2735 |
| *HFX_2735*-R | TATCTAGAGATATCCGGCGACGACGATACAACTC |
| *HFX_2424*-F | CCGAGTTAGGAGATGGGATCCTGATTCGAATGACTTCCGTCGGCATCGA | Amplification of *HFX_2424* gene inserted into pWLR to construct pW2424 |
| *HFX_2424*-R | CGCACACAAGAAAACGGTACCCTACTCGACGTACTCGTAGC |
| *HFX_2609*-F | CCGAGTTAGGAGATGGGATCCTGATTCGAATGACAGACGCTGCGTCTCT | Amplification of *HFX_2609* gene inserted into pWLR to construct pW2609 |
| *HFX_2609*-R | CGCACACAAGAAAACGGTACCTTACCGACCGAGGTCGGCGT |
|  |  |  |
|  |  |  |
| **Primers** | **Sequence (5'→3')** | **Usage** |
| *HFX_2519*-F | CCGAGTTAGGAGATGGGATCCTGATTCGAATGAGCGACGCACAGGCCGG | Amplification of *HFX_2519* gene inserted into pWLR to construct pW2519 |
| *HFX_2519*-R | CGCACACAAGAAAACGGTACCTTATTCGAAGTCACGGCGCA |
| *HFX_2550*-F | TTGGATCCTGAGAGCTCATGGATGATAGCCACATCGT | Amplification of *HFX_2550* gene inserted into pWLR to construct pW2550 |
| *HFX_2550*-R | TTTCTAGACTACGTCGAGAGGTCGTCGC |
| *HFX_2547*-F | TTGGATCCTGAATGCTCAACGAATCACAGGT | Amplification of *HFX_2547* gene inserted into pWLR to construct pW2547 |
| *HFX_2547*-R | TTTCTAGAGAGCTCTCAGTCCGAACCCCATCGAC |
| *crtB*-in-1 | TTGCATGCCGATGACCGTCGTACATGAG | Amplification of homologous arm sequences of *crtB_hm_* and inserted into pHFX to construct pHFXB |
| *crtB*-in-2 | TCGACACCATTAGATCGTATACCGCGTACCGTCCC |
| *crtB*-in-3 | GGTACGCGGTATACGATCTAATGGTGTCGAAGGGA |
| *crtB*-in-4 | TAGGTACCCGGAGGAAGTTCGACAGTTG |
| *HFX_2549*-K1 | TTGAGCTCACGGAGACATGGGAGAAGGG | Amplification of homologous arm sequences of *HFX_2549* inserted into pHFX to construct pHFX2549K |
| *HFX_2549*-K2 | AGCTGCGTTCGCGAGGGCGAGGAAGAAGTACGCGA |
| *HFX_2549*-K3 | TACTTCTTCCTCGCCCTCGCGAACGCAGCTCCCGA |
| *HFX_2549*-K4 | TTGCATGCCACCACGTCCATGGCGAGGA |
| *phaEC*-K1 | CAGGTCGACTCTAGAGGATCCACAAGCCAACCCATTCACGC | Amplification of homologous arm sequences of *phaEC* and inserted into pHFX construct pHFXPK |
| *phaEC*-K2 | ACGTCGAAAAAACGAGGGCATATACTCTCGGGCGG |
| *phaEC*-K3 | CGAGAGTATATGCCCTCGTTTTTTCGACGTGAAAA |
| *phaEC*-K4 | AACCAGTACCGGTAAGGTACCAAAACCGACCAGTCCTGCGT |
| **Primers** | **Sequence (5'→3')** | **Usage** |
| *HAH_2563*-F | TTGGATCCTGATTCGAATGCACTCCGATAACATCCA | Amplification of *HAH_2563* gene inserted into pWLR to construct pWHA2563 |
| *HAH_2563*-R | TTTCTAGATCATCCACGCCACGCGGGTT |
| *OE_3093*-F | CCGAGTTAGGAGATGGGATCCTGATTCGAATGGTCTCACCGGAACACCT | Amplification of *OE_3093R* gene inserted into pWLR to construct pWOE3093 |
| *OE_3093*-R | CGCACACAAGAAAACGGTACCTCAGTCCGCCCGCGTGGGCT |
| *HAH_1058* | CCGAGTTAGGAGATGGGATCCTGATTCGAATGAGTGACTTGTCCGGTGA | Amplification of *HAH_1058* gene inserted into pWLR to construct pWHA1058 |
| *HAH_1058* | CGCACACAAGAAAACGGTACCTCAGGCGATATCCTCGATGA |
| *OE_3381*-F | CCGAGTTAGGAGATGGGATCCTGATTCGAATGGACGAACTCGCGGGGAC | Amplification of *OE_3381R* gene inserted into pWLR to construct pWOE3381 |
| *OE_3381*-R | CGCACACAAGAAAACGGTACCTCAGCGCGCGTCGGCGAGCA |
| *HAH_1058*-in1 | CAGGTCGACTCTAGAGGATCCGACCGACGACACGGTCGCAC | Amplification of the homologous arm sequences of *HFX_2549* and *HAH_1058* gene inserted into pHFX to construct pHF1058 |
| *HAH_1058*-in2 | CTCAGACCTCTTCATCTCAT |
| *HAH_1058*-in3 | ATGAAGAGGTCTGAGATCTAATGGTGTCGAAGGGA |
| *HAH_1058*-in4 | TCAGGCGATATCCTCGATGA |
| *HAH_1058*-in5 | GAGGATATCGCCTGACTCGCGAACGCAGCTCCCGA |
| *HAH_1058*-  in6 | AACCAGTACCGGTAAGGTACCGCCGGGGTCGAGCACCACGT |
| **Primers** | **Sequence (5'→3')** | **Usage** |
| *OE_3381*-in1 | CAGGTCGACTCTAGAGGATCCGACCGACGACACGGTCGCAC | Amplification of the homologous arm sequences of *HFX_2549* and *OE_3381R* gene inserted into pHFX to construct pHF3381 |
| *OE_3381*-in2 | CTCAGACCTCTTCATCTCAT |
| *OE_3381*-in3 | ATGAAGAGGTCTGAGATCTAATGGTGTCGAAGGGA |
| *OE_3381*-in4 | TCAGCGCGCGTCGGCGAGCA |
| *OE_3381*-in5 | TCAGCGCGCGTCGGCGAGCA |
| *OE_3381*-in6 | AACCAGTACCGGTAAGGTACCGCCGGGGTCGAGCACCACGT |
| B6I6-1 | CAGGTCGACTCTAGAGGATCCGACCGACGACACGGTCGCAC | Amplification of homologous arm sequences up-stream and down-stream of *HFX_2549, HAH_1058* and *HAH_2563* genes inserted  into pHFX to construct pB6I6 |
| B6I6-2 | CTCAGACCTCTTCATCTCAT |
| B6I6-3 | ATGAAGAGGTCTGAGATCTAATGGTGTCGAAGGGA |
| B6I6-4 | AAAAAAAATCATCCACGCCACGCGGGTT |
| B6I6-5 | TGGATGATTTTTTTTATCTAATGGTGTCGAAGGGA |
| B6I6-6 | TCAGGCGATATCCTCGATGA |
| B6I6-7 | GAGGATATCGCCTGACTCGCGAACGCAGCTCCCGA |
| B6I6-8 | AACCAGTACCGGTAAGGTACCGCCGGGGTCGAGCACCACGT |
| BHI6-1 | CAGGTCGACTCTAGAGGATCCGACCGACGACACGGTCGCAC | Amplification of homologous arm sequences up-stream and down-stream of *HFX_2549*, *HAH_1058* and *OE_3093R* genes inserted into pHFX to construct pBHI6 |
| BHI6-2 | CTCAGACCTCTTCATCTCAT |
| BHI6-3 | ATGAAGAGGTCTGAGATCTAATGGTGTCGAAGGGA |
| BHI6-4 | AAAAAAAATCAGTCCGCCCGCGTGGGCT |
| BHI6-5 | GGACTGATTTTTTTTATCTAATGGTGTCGAAGGGA |
| BHI6-6 | TCAGGCGATATCCTCGATGA |
| BHI6-7 | GAGGATATCGCCTGACTCGCGAACGCAGCTCCCGA |
| BHI6-8 | AACCAGTACCGGTAAGGTACCGCCGGGGTCGAGCACCACGT |
| pyrF-F | CAGGTCGACTCTAGAGGATCCCAGCGACAAATACGGCCGTG | Amplification of *pyrF* gene with 500-bp up-stream and down-stream sequences |
| pyrF-R | AACCAGTACCGGTAAGGTACCGGGATGAGCCGAAAGAAGGC |
| **Primers** | **Sequence (5'→3')** | **Usage** |
| 7S-Q1 | CACCGATGAACCGCGCAAAC | Quantification of 7S rRNA |
| 7S-Q2 | GACTAGGTCGGGCAGTTAGG |  |
| 50B-Q1 | AGACAGGTGACCCGGTGTTG | Quantification of *crtB_hm_* mRNA |
| 50B-Q2 | CATCACGGCGGTCATCATGC |  |
| B6-Q1 | TCCAGCACGAGCAACTGGAG | Analysis of *crtB_ha_* transcription by RT-PCR |
| B6-Q2 | GGTCCTCGAACGTCTCGTAG |  |
| BH-Q1 | GTCCTGTACGCGTTCTTCCG | Analysis of *crtB_hs_* transcription by RT-PCR |
| BH-Q2 | TGGCGTCGATGAACGTGGTG |  |
| I6-Q1 | GGTATCTGATGCCCGACGTG | Analysis of *crtI_ha_* transcription by RT-PCR |
| I6-Q2 | CGCGAGGTAGTCTTCGAACG |  |

All strains are cultured in shake flasks with MG medium for 7 days. Data represents the Mean ± SD of three independent experiments.


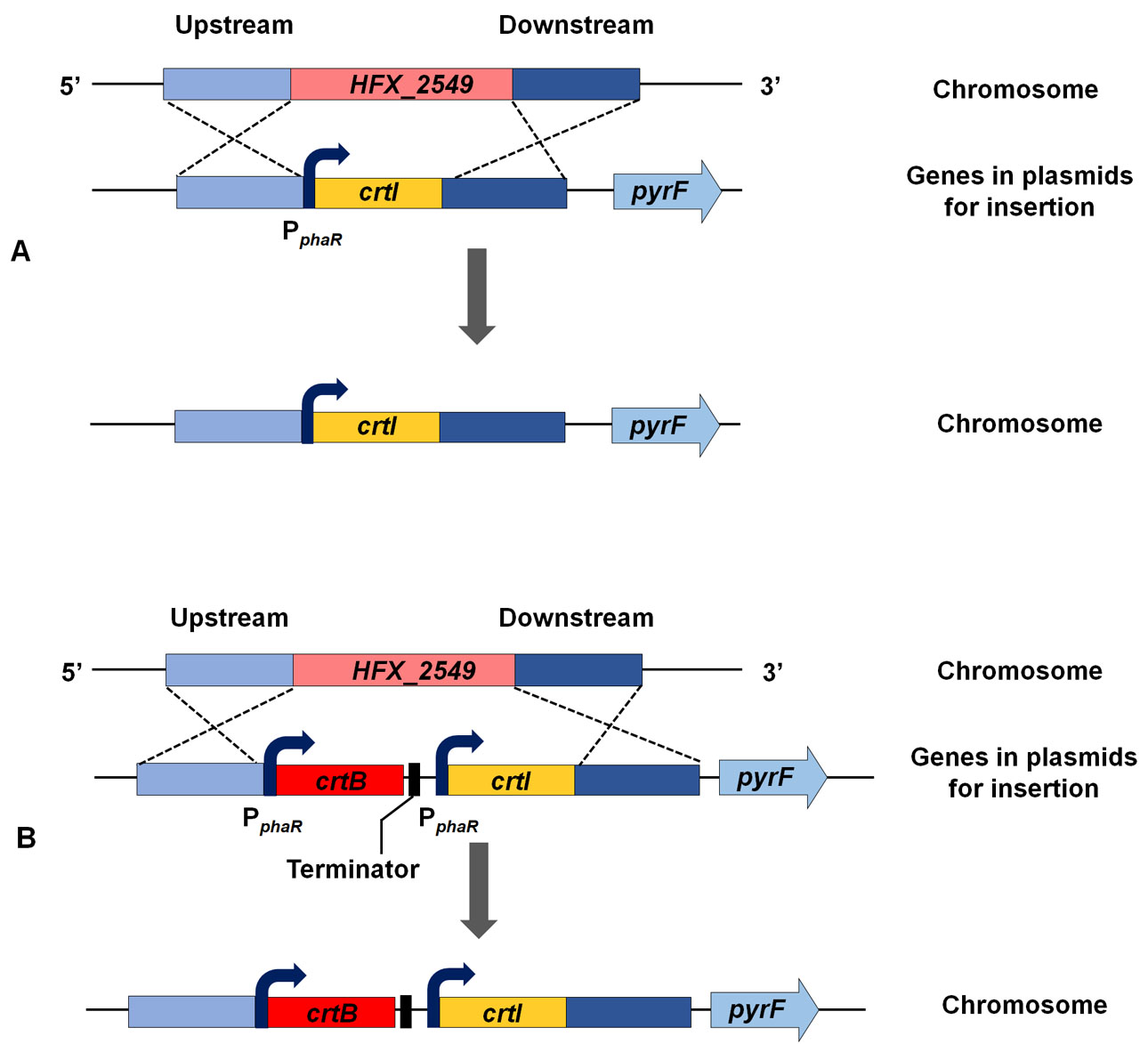


**Supplementary Figure S1.** Strategies for heterologous *crtB* and *crtI* integration in the chromosome of *H. mediterranei*. **(A)** Heterologous *crtI* insertion by homologous recombination. **(B)** Heterologous *crtB* and *crtI* co-insertion by homologous recombination.


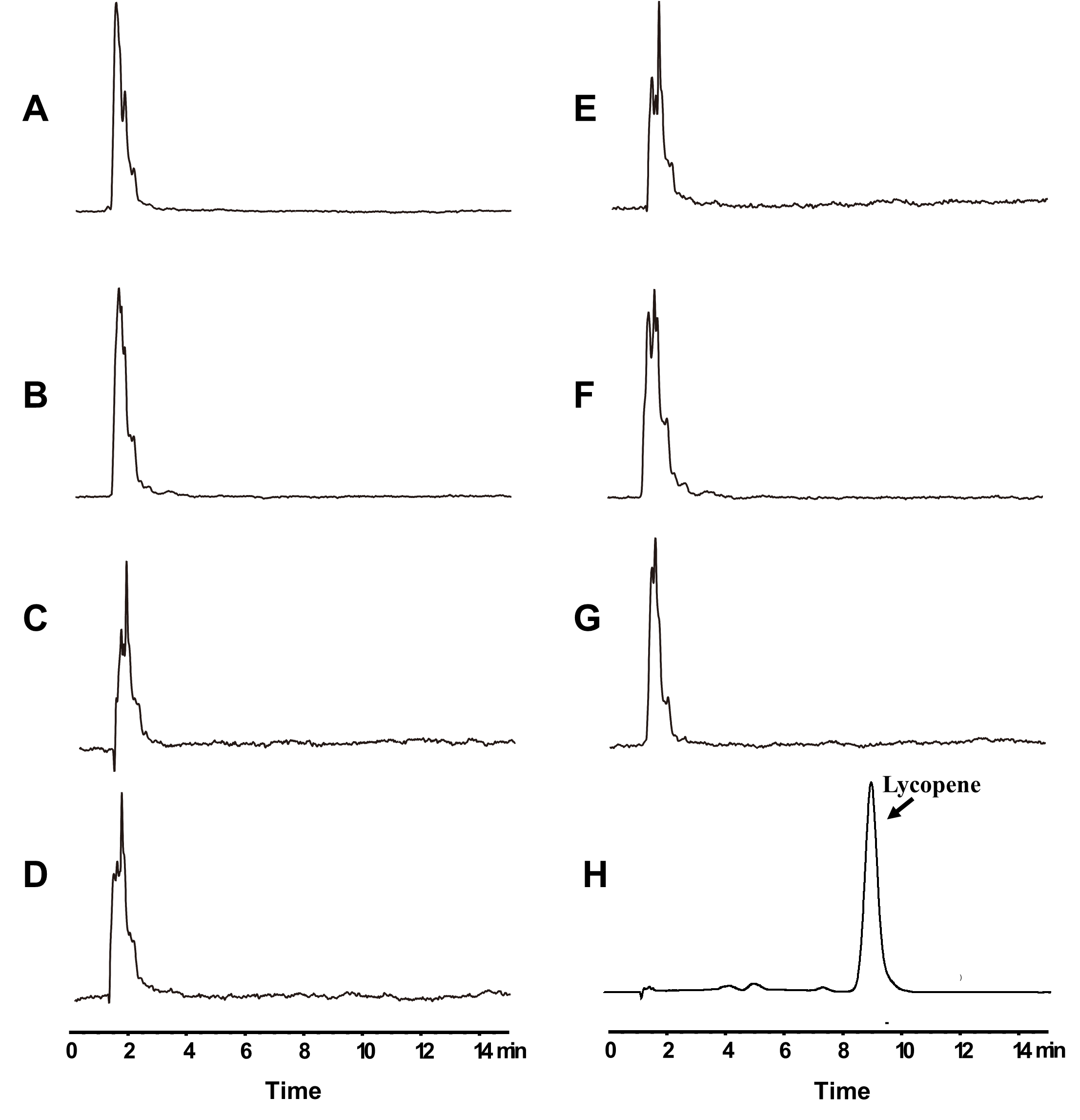


**Supplementary Figure S2.** HPLC analysis of the lycopene produced by DF50Δeps strains with different gene overexpression. **(A)** DF50-2424; **(B)** DF50-2609; **(C)** DF50-2773/4; **(D)** DF50-1486; **(E)** DF50-2519; **(F)** DF50-2735; **(G)** DF50-2550; **(H)** Lycopene standard.

**A**


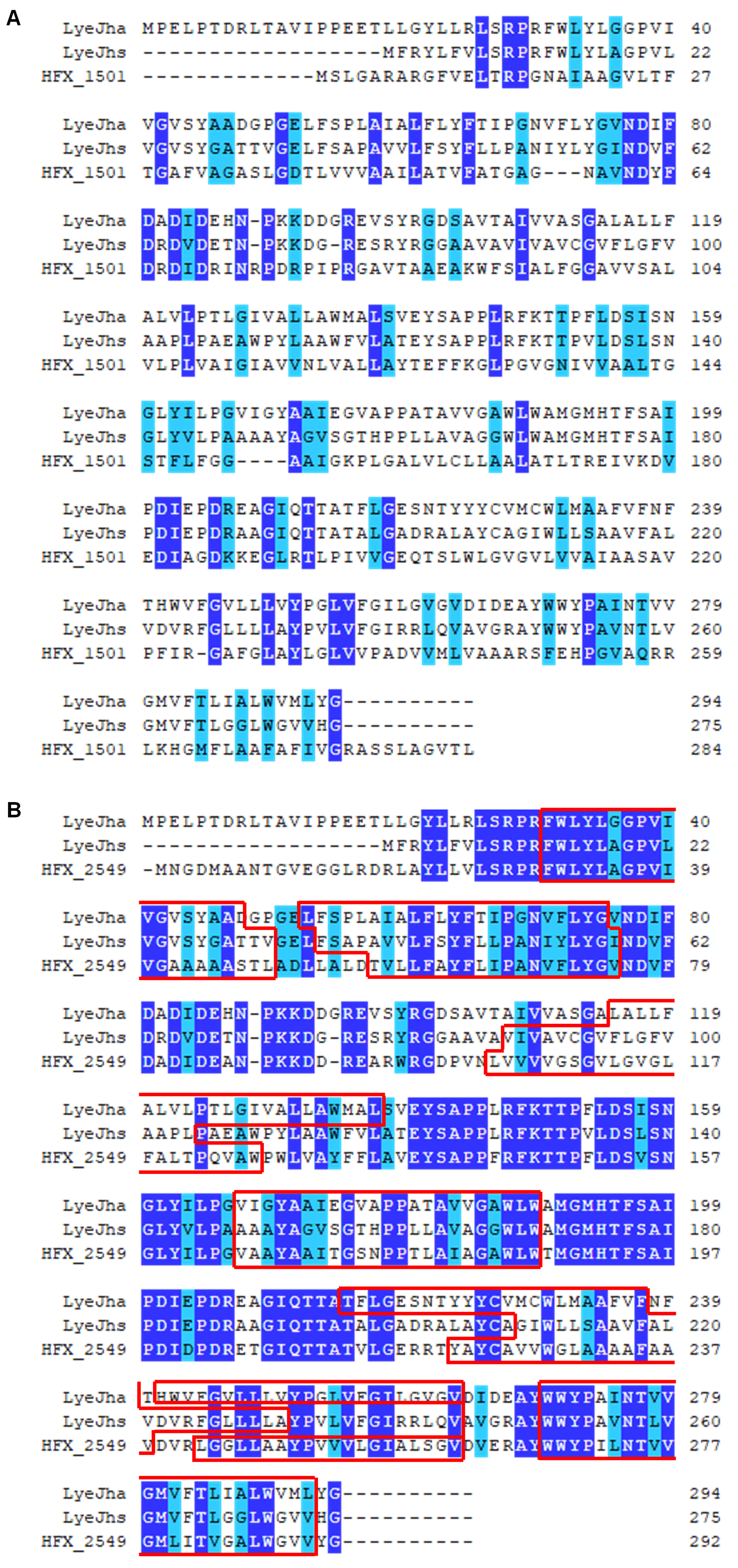


**Supplementary Figure S3.** Alignment of the protein sequences of LyeJ from haloarchaea. **(A)** Multiple alignments of amino acid sequences of the putative LyeJ. The conserved residues were highlighted in dark blue, and the residues identical in two of the three were in lightly blue. **(B)** Predictions of transmembrane helices of the putative lycopene elongase encoded by *HFX_2549* in *H. mediterranei*. The transmembrane segments predicted by TMHMM (Krogh et al., 2001) were outlined in red.


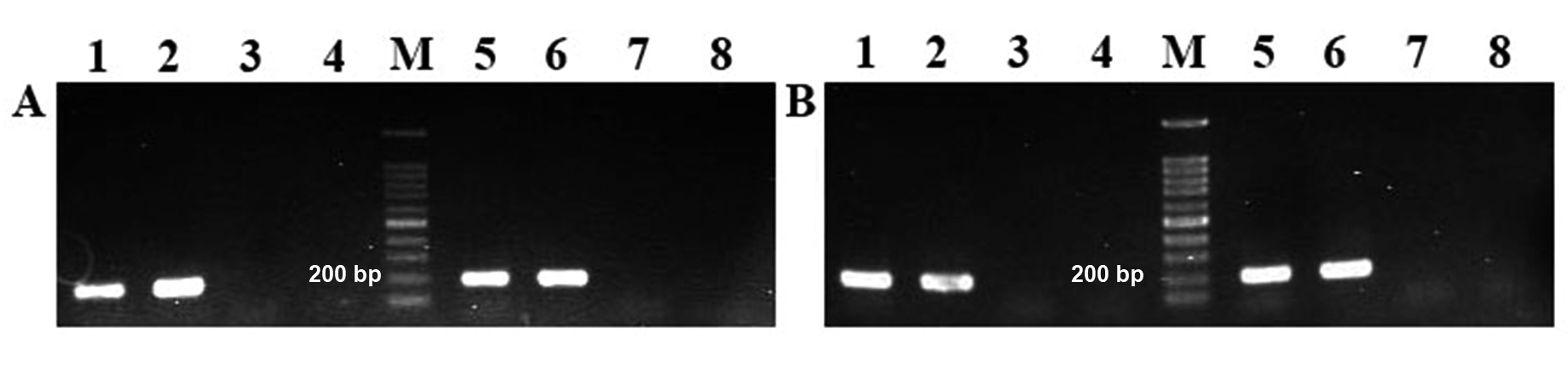


**Supplementary Figure S4.** Expression of *crtI* and *crtB* from *H. hispanica and H. salinarum* in 50B6I6 and 50BHI6. **(A)** RT-PCR analysis of *crtB_ha_* and *crtI_ha_* in 50B6I6. Line 1, the cDNA reverse transcribed from the RNA extracted from 50B6I6 was the template; Line 2, plasmid pWHA2563 was the template; Line 3, the genome of DF50Δeps was the template; Line 4, RNA extracted from 50B6I6 was the template; M, 100-bp DNA marker; Line 5, the cDNA reverse transcribed from the RNA extracted from 50B6I6 was the template; Line 6, plasmid pWHA1058 was the template; Line 7, the genome of DF50Δeps was the template; Line 8, RNA extracted from 50B6I6 was the template. **(B)** RT-PCR analysis of *crtB_hs_* and *crtI_ha_* in 50BHI6. Line 1, the cDNA reverse transcribed from the RNA extracted from 50BHI6 as template; Line 2-4, negative control similar to **(A)**; M, 100-bp DNA marker; Line 5, reverse-transcribed cDNA of the RNA extracted from 50BHI6 was the template; Line 6, same to **(A)**; Line 7-8, negative control by using the RNA from DF50Δeps or 50BHI6 as the template.

# References

Cai, S., Cai, L., Liu, H., Liu, X., Han, J., Zhou, J., et al. (2012). Identification of the haloarchaeal Phasin (PhaP) that functions in polyhydroxyalkanoate accumulation and granule formation in *Haloferax mediterranei*. *Appl. Environ. Microbiol.* 78, 1946-1952. doi: 10.1128/AEM.07114-11.

Krogh, A., Larsson, B., von Heijne, G., and Sonnhammer, E.L.L. (2001). Predicting transmembrane protein topology with a hidden Markov model: Application to complete genomes. *J. Mol. Biol*. 305, 567-580. doi: 10.1006/jmbi.2000.4315.

Liu, H.L., Han, J., Liu, X.Q., Zhou, J., and Xiang, H. (2011). Development of *pyrF*-based gene knockout systems for genome-wide manipulation of the archaea *Haloferax mediterranei* and *Haloarcula hispanica*. *J. Genet. Genomics.* 38, 261-269. doi: 10.1016/j.jgg.2011.05.003.

Palmer, B.R., and Marinus, M.G. (1994). The *dam* and *dcm* strains of *Escherichia coli*-a review. *Gene* 143, 1-12. doi: 10.1016/0378-1119(94)90597-5.

Sambrook, H.C. (1989). Molecular cloning : a laboratory manual. Cold Spring Harbor, NY.

Zhao, D., Cai, L., Wu, J., Li, M., Liu, H., Han, J., et al. (2013). Improving polyhydroxyalkanoate production by knocking out the genes involved in exopolysaccharide biosynthesis in *Haloferax mediterranei*. *Appl. Microbiol. Biotechnol.* 97, 3027-3036. doi: 10.1007/s00253-012-4415-3.
